# The Influence of Intervertebral Disc Microenvironment on the Biological Behavior of Engrafted Mesenchymal Stem Cells

**DOI:** 10.1155/2022/8671482

**Published:** 2022-11-07

**Authors:** Jing Zhang, Wentao Zhang, Tianze Sun, Jinzuo Wang, Ying Li, Jing Liu, Zhonghai Li

**Affiliations:** ^1^Department of Orthopedics, First Affiliated Hospital of Dalian Medical University, Dalian, 116011 Liaoning, China; ^2^Stem Cell Clinical Research Centers, National Joint Engineering Laboratory, The First Affiliated Hospital of Dalian Medical University, Dalian, 116021 Liaoning, China

## Abstract

Intervertebral disc degeneration is the main cause of low back pain. Traditional treatment methods cannot repair degenerated intervertebral disc tissue. The emergence of stem cell therapy makes it possible to regenerate and repair degenerated intervertebral disc tissue. At present, mesenchymal stem cells are the most studied, and different types of mesenchymal stem cells have their own characteristics. However, due to the harsh and complex internal microenvironment of the intervertebral disc, it will affect the biological behaviors of the implanted mesenchymal stem cells, such as viability, proliferation, migration, and chondrogenic differentiation, thereby affecting the therapeutic effect. This review is aimed at summarizing the influence of each intervertebral disc microenvironmental factor on the biological behavior of mesenchymal stem cells, so as to provide new ideas for using tissue engineering technology to assist stem cells to overcome the influence of the microenvironment in the future.

## 1. Introduction

Low back pain (LBP) is a common health concern worldwide. Studies have shown that the prevalence of LBP ranges between 1.4% and 20%, which is the main reason for years lived with disability counts and puts a heavy economic burden on the patients and society [1–3]. The diseases that present with symptoms of LBP are mainly spinal degenerative diseases, such as discogenic LBP and lumbar disc herniation [4], and their occurrence is closely related to intervertebral disc (IVD) degeneration (IDD) [5, 6].

Due to the varying severity of clinical manifestations in patients, step-by-step therapy is often used in clinical practice. Most patients tend to opt for conservative treatments due to mild symptoms and short course of the disease, including bed rest, oral painkillers, and functional exercises. Interventional treatments, such as epidural injections and percutaneous intradiscal therapies, are generally performed if conservative treatments fail [7, 8]. With severe symptoms or ineffective conservative and interventional treatments, surgery is often recommended. Although surgical treatments can effectively relieve pain, they may cause complications such as infection, nerve damage, large blood vessel damage, and adjacent segment degeneration due to improper operation or care, which further damage the body of the patients [9, 10]. In addition, neither conservative treatments, interventional treatments, nor surgical treatments can repair the degenerated IVD tissue. Traditional treatments are in a dilemma, and a new treatment is urgently needed to induce repair of degenerated disc tissue.

In recent years, with the successful application of stem cell therapy in neurological diseases, cardiovascular diseases, diabetes, and other fields [11–13], researchers have begun to explore the therapeutic effect of stem cells in IDD. Stem cells come from a wide range of sources, among which mesenchymal stem cells (MSCs) are the most studied. There are several studies that have shown disc height restoration, T2-weighted signal intensity on MRI, improved histology, extracellular matrix (ECM) gene expression, and pain relief after transplantation of MSCs in animal models and preliminary human clinical trials [14–19]. However, the optimal type of MSCs for the treatment of intervertebral disc degeneration has not yet been determined. In addition, IVD is an avascular tissue with a harsh and complex internal microenvironment, which has been shown to have an impact on the biological behavior of the implanted MSCs, which in turn affects the therapeutic effect [20, 21]. Some studies even reported that MSCs did not demonstrate superior benefits for IDD compared with other therapies, and microenvironmental factors were an important reason for the failure of stem cell repair [22, 23]. These contradictory results suggest that the successful translation of stem cell transplantation for IDD into clinical efficacy remains a formidable challenge. Therefore, in order to accurately utilize tissue engineering technology to improve the therapeutic effect of MSCs in the treatment of IDD, we need to better understand the influence of the internal microenvironment of the IVD on the biological behavior of the implanted stem cells. This review is aimed at outlining the advantages and disadvantages of the different types of MSCs for IDD treatment and focuses on the impact of various microenvironmental factors on the biological behavior of MSC viability, proliferation, migration, and chondrogenic differentiation.

## 2. IDD and MSCs

### 2.1. The Basic Structure of the IVD

The IVD is composed of an outer annulus fibrosus (AF), a nucleus pulposus (NP) in the middle, and cartilage endplates (CEPs) at the upper and lower ends. The AF is arranged in layers with the NP as the center. The outer layer is composed of collagen-I, and collagen-II increases gradually from outside to inside, and all the collagen-II reaches the NP [24]. NP is the main structure of IVD, which is mainly composed of NP cells and ECM. The main components of ECM are collagen-II, proteoglycans, and other matrix proteins, the first two of which are two markers of chondrogenesis [25]. Proteoglycans are a special class of glycoproteins consisting of a core protein and one or more glycosaminoglycans, with multiple proteoglycans linked to hyaluronic acid (HA) chains to form aggrecans. Aggrecan provides a high-level charge density that maintains water within the NP by creating a high osmotic pressure [26]. Since the NP is highly hydrated, the IVD tissue has a high degree of elasticity and is able to withstand and buffer normal spinal biological stress. In the absence of a normal ECM, disc degeneration will occur.

### 2.2. Application of Endogenous and Exogenous MSCs in the Treatment of IDD

**Figure 1:**
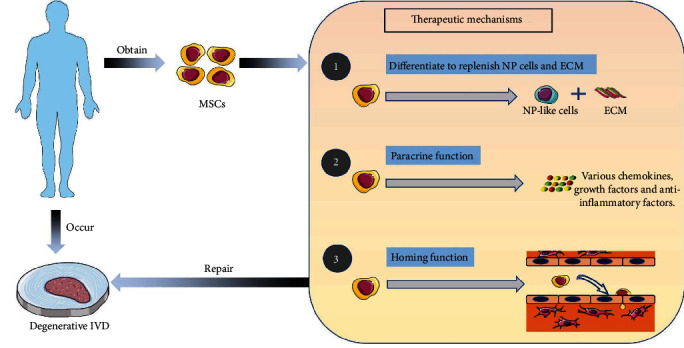
The main mechanisms of MSCs in the treatment of IDD. ECM: extracellular matrix; IVD: intervertebral disc; MSCs: mesenchymal stem cells; IDD: intervertebral disc degeneration.

The most important pathophysiological changes in IDD are reduced NP cells and enhanced ECM catabolism. Therefore, it becomes a reasonable therapeutic strategy to replenish lost NP cells and ECM by implanting MSCs into degenerative IVD tissues. NP cells are chondrocyte-like cells, and there is no specific and generally accepted marker to differentiate them from chondrocytes [21, 27]. MSCs are cells with self-replication, renewal, multidirectional differentiation, and paracrine potential, which mainly achieve the purpose of treating IDD through two major mechanisms. On the one hand, MSCs have the ability to differentiate into chondroblasts, so they can proliferate and differentiate into NP cells and synthesize ECM after implantation [28, 29]. On the other hand, MSCs have paracrine capacity. They can not only secrete a variety of growth factors to increase the activity of resident IVD cells and promote the synthesis of ECM but also secrete certain anti-inflammatory cytokines to regulate the inflammatory response in degenerated IVD and delay the process of IDD [30–32]. Since the process of transplanting MSCs into degenerative IVDs is an invasive procedure, researchers have increasingly focused on the possibility of homing exogenous and endogenous MSCs into IVDs [33]. The homing of MSCs refers to a process in which cells are recruited from their original niche to injured or pathological tissue, which can be induced by a variety of growth factors and chemokines [33, 34]. However, this endogenous repair strategy for IDD is still in the preclinical research stage and requires further research (Figure 1).

There are many types of MSCs that are used for IDD treatment. According to different tissue sources, MSCs can be divided into two categories: endogenous and exogenous MSCs. Endogenous MSCs refer to IVD-derived stem cells isolated and cultured from normal and degenerative IVD tissue. According to the different localization, endogenous MSCs can be divided into three types: NP-derived MSCs (NP-MSCs), AF-derived MSCs (AF-MSCs), and CEP-derived MSCs (CE-MSCs) [35, 36]. Due to the basic characteristics of MSCs, most endogenous MSCs express MSC-like surface markers including CD73, CD90, and CD105 on the cell surface but do not express CD14, CD19, CD34, CD45, and HLA-DR [36–40]. Some researchers believe that stem cells may have their own specific expression of cell surface markers. For example, stromal cell antigen-1 (STRO-1) may be a specific cell surface marker of CE-MSCs [39, 41], and tyrosine kinase receptor-2 (Tie-2) and disialoganglioside-2 (GD-2) can be used as specific cell surface markers of NP-MSCs [42]. However, further research is needed on the cell surface specific markers of endogenous MSCs. In addition, endogenous MSCs have the ability of osteogenic, adipogenic, and chondrogenic differentiation. In terms of chondrogenic differentiation, some studies have shown that CE-MSCs have stronger differentiation ability, followed by AF-MSCs and NP-MSCs that are lower [36, 43]. The most common types of exogenous MSCs are bone marrow-derived MSCs (BMSCs) and adipose-derived MSCs (AD-MSCs), a few are umbilical cord-derived MSCs (UC-MSCs), and there are other rare types of MSCs, such as placental-derived MSCs (PMSCs), amniotic fluid-derived MSCs (AF-MSCs), amniotic membrane-derived MSCs (AMSCs), synovial-derived MSCs (SMSCs), and peripheral blood-derived MSCs (PB-MSCs). These different tissue-derived MSCs have their own advantages and disadvantages when applied to IDD treatment (Table 1) [36, 40, 44–51].

Besides the direct application of MSCs for the treatment of IDD, some researchers have also focused on the indirect use of MSCs, among which extracellular vesicles (EVs) have attracted attention [52]. EVs are lipid bilayer-surrounded vesicles that are incapable of replicating and do not contain a functional nucleus. In terms of their biogenesis, release pathways, size, content, and function, EVs can be divided into three main subtypes: exosomes, microvesicles (MV), and apoptotic bodies [53]. Exosomes are paracrine cell communication vesicles with a diameter ranging from 30 to 120 nm, which can carry a variety of biologically active molecules such as mRNA, microRNA (miRNA), protein, and lipid, and have great potential in cell-free therapy [54–56]. Compared with other cell types, MSCs can produce more exosomes through the paracrine pathway [57]. The results have shown that MSC-Exos can delay or even reverse the degenerative process of IVD by maintaining ECM homeostasis, inhibiting apoptosis of NP cells, antioxidative, and anti-inflammatory effects, although the exact underlying mechanisms have not been fully elucidated [58–61]. MSC-derived exosomes (MSC-Exos) have unique advantages in the treatment of IDD when compared with MSCs. Firstly, MSC-Exos do not have the associated risks of immunoreactivity and differentiation into unwanted cells, since they are not cells [62]. Secondly, genetic modification of exosomes to express specific ligands allows them to serve as vehicles for targeted drug delivery [63, 64]. Although MSC-Exos are a promising treatment for IDD, many challenges remain before their clinical application. For example, there are currently no standardized protocols for exosomes isolation, and detection methods with high sensitivity and specificity for the therapeutic efficacy of MSC-Exos are lacking [65, 66]. Furthermore, the optimal route of administration of MSC-Exos remains unclear [67, 68]. These challenges remain to be addressed by further research.

## 3. Influence of the IVD Microenvironmental Factors on MSCs

**Figure 2:**
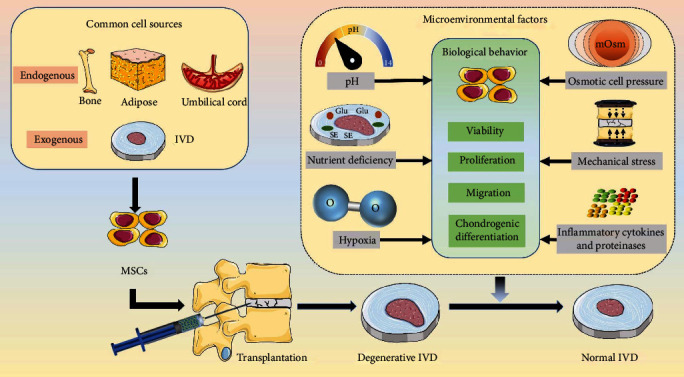
Schematic diagram of the influence of six intervertebral disc microenvironmental factors on the biological behavior of the implanted mesenchymal stem cells.

Physiologically, the IVD microenvironment is characterized by hypoxia, nutrient deficiency, acidity, hypertonicity, and mechanical loading. During the degeneration of the IVD, its internal microenvironment further deteriorates, and mechanical overload, inflammatory cytokines, and protease accumulation also occur [69, 70]. These microenvironmental factors not only affect the resident IVD cells but also have an impact on the biological behavior of the implanted MSCs. The effects of different microenvironmental factors on MSCs are not completely consistent, and the tolerance of the different types of MSCs to the IVD microenvironment is also different. Next, the effects of the following six microenvironmental factors on the biological behavior of engrafted MSCs are described (Figure 2).

### 3.1. Hypoxia

The partial pressure of oxygen in normal adult IVD can be reduced from 10% in the lateral annulus region to 1% in the central region and is always in a hypoxic state [71]. As IVD degenerates, its partial pressure of oxygen may drop even lower. In general, the therapeutic potential of MSCs is investigated under conventional normoxic conditions. Therefore, MSCs transplanted into degenerative discs are in a hypoxic environment. Compared with normoxic conditions, hypoxia can promote the viability, proliferation, and migration of BMSCs [72–75] and can also inhibit senescence and maintain the stemness of BMSCs through downregulation of E2A-p21 by HIF-1*α*-Twist pathway [73, 74]. However, other researchers observed that hypoxia inhibited the migration of BMSCs through hypoxia-inducible factor-1*α* (HIF-1*α*) and RhoA-mediated pathways [76].

In addition, multiple studies have demonstrated that hypoxia can reduce the inhibitory effect of interleukin- (IL-) 1*β* on the chondrogenic differentiation of BMSCs [77], improve the quality of the generated ECM [77, 78], and promote the differentiation of BMSCs to the NP-like phenotype [78–80]. HIF-1*α* is a key mediator of the beneficial effects of hypoxic environment on chondrogenic differentiation of MSCs [81]. These results support the enhancement of the ability of BMSCs to repair degenerative IVD by hypoxic preconditioning [75, 82].

Although hypoxic environment has also been shown to promote the migration [83] and chondrogenic differentiation [83–85] of AD-MSCs, the results of hypoxia in terms of their viability and proliferation are still controversial. Most studies have shown that hypoxia enhanced the viability and proliferation rate of AD-MSCs [83, 86–88]. Compared with BMSCs, AD-MSCs had a higher tolerance to hypoxia, serum-free and oxidative stress condition [89]. Further research by a team found that HIF-1*α* promotes the proliferation of AD-MSCs by interacting with basic fibroblast growth factor and vascular endothelial growth factor under hypoxic conditions [90]. However, there are also studies showing conflicting results. Chung et al. [91] found that hypoxia at 1% and 5% oxygen tension negatively affected both the proliferation rate and osteogenic differentiation of AD-MSCs. The results of Li et al. [44] showed that hypoxia with 2% oxygen tension inhibited the viability and proliferation of AD-MSCs and NP-MSCs but promoted the chondrogenic differentiation of cells, and NP-MSCs exhibited more potent biological activity. He et al. [92] found that hypoxia stimulates autophagy via the HIF-1*α* signaling pathway, thereby increasing NP-MSC resistance to hydrostatic pressure. These findings highlight the beneficial role of hypoxia in stem cell-based IVD regeneration, thereby providing a promising therapeutic target for IDD therapy. In addition, other studies have also observed that hypoxia promotes the proliferation and migration of UC-MSCs [93]. The controversies about the effect of hypoxia on the viability and proliferation of AD-MSCs may be caused by the heterogeneity of different research protocols, medium components, oxygen tension and donors. Further studies are needed to explore and clarify.

In addition to the above types of MSCs, some researchers have also explored the effect of hypoxia on other rare types of MSCs. Some studies have shown that compared with normoxic environment, hypoxic environment promoted the early proliferation and differentiation of PMSCs into NP-like cells [94–97] and enhanced the migration ability of PMSCs by increasing the activity of matrix metalloproteinase- (MMP-) 2[96]. Other studies have also observed the effect of hypoxia on AF-MSCs, SMSCs, and PB-MSCs and found that hypoxia enhanced the proliferation potential and stemness characteristics of AF-MSCs [98, 99] and PB-MSCs [100] and promoted the chondrogenic differentiation of SMSCs [101]. These results suggest that hypoxic culture conditions may serve as an effective strategy to maintain the function of MSCs (Table 2).

### 3.2. Nutrient Deficiency

IVD cells are supplied with essential nutrients by the diffusion from the blood supply through mainly the CEPs and disc tissue, and one of the key nutrients is glucose. A normal IVD is in an IVD-like low-glucose environment, with glucose concentrations dropping from about 5 mM in the outer AF to about 1 mM in the central NP [102]. After IDD, the glucose concentration in the NP is further decreased due to various factors including calcification of the CEPs and reduced blood supply to the vertebral body. Glucose concentrations in human degenerated NP average 0.603 ± 0.108 mM [71], and 0.5 mM is the minimum glucose level required for IVD cell survival [103]. The researchers explored the effect of glucose concentration on the biological behavior of BMSCs [104] and AD-MSCs [105]. They found that IVD-like low glucose (5.5 mM), although mildly inhibited the viability of MSCs, promoted aggrecan expression and resulted in a small increase in proliferation compared to standard conditions (25 mM) [104, 105]. Some researchers also observed that the limited glucose condition inhibited the viability of NP-MSCs [106]. Hypoxia and nutritional deficiency often coexist in degenerated discs. Farrell et al. [107] found that IVD-like low glucose (5.5 mM) limited the viability of BMSCs and that there was heterogeneity in the response of the MSC population to metabolic stressors. Naqvi and Buckley [108] investigated the effect of different glucose concentrations on BMSCs under 5% hypoxia and found that IVD-like low glucose (5 mM) increased the accumulation of sulphated glycosaminoglycans (sGAG) and collagen. In contrast, too low glucose concentration (1 mM) promoted BMSC death and inhibited proliferation and the accumulation of sGAG and collagen [108]. These results suggest that IVD-like low-glucose conditions may be a positive factor for MSC transplantation in the treatment of IDD, while too low concentrations of glucose can have significant negative effects. Recently, Yang et al. [109] further studied and found that oxygen-glucose deprivation not only significantly inhibited the viability and adipogenesis of AD-MSCs but also inhibited cell proliferation and migration. Inhibition of receptor-interacting protein kinase-3 (RIP3) increased the viability, proliferation, and migration of AD-MSCs under oxygen-glucose deprivation and reduced unstable neovascularization and inhibited inflammatory responses [109].

In addition to glucose, serum is also an important nutrient for IVD cells. Parker et al. [110] found that both very low-serum and serum-free conditions maintained the chondrogenic differentiation ability of AD-MSCs. Since this study was conducted under normoxic conditions, Wan Safwani et al. [111] conducted a more in-depth study to clarify the exact effects of serum deprivation and hypoxia on MSCs. They found that serum deprivation inhibited the viability, proliferation, and adipogenic potential of AD-MSCs regardless of hypoxia but increased aggrecan gene expression levels, which in turn enhanced the chondrogenic differentiation of AD-MSCs [111]. Takahashi et al. [89] found that although serum deprivation inhibited the viability of MSCs, AD-MSCs were more tolerant to hypoxia, oxidative stress, and serum-free condition than BMSCs. In addition, hypoxia [112, 113] or glucose deprivation [114–116] under serum deprivation conditions can significantly reduce the viability and proliferation of MSCs and increase the production of reactive oxygen species and apoptosis. Berberine protects MSCs from autophagy and apoptosis through the AMP-activated protein kinase (AMPK) signaling pathway [113, 114]. Similarly, an appropriate concentration of acetyl-L-carnitine (ALC) can protect MSCs from nutrient deprivation-induced injury by enhancing the expression of survival signals and reducing the expression of death signals [115, 116]. Some studies have reported the impact of hypoxia and serum deprivation on rare type MSCs. Huang et al. [117, 118] found that hypoxia significantly enhanced the proliferation of PMSCs, while serum deprivation inhibited the growth of PMSCs. In addition, hypoxia and serum deprivation did not induce apoptosis of PMSCs, which may be related to the high expression of BCL-2 in serum deprivation [117]. These results indicate that PMSCs may be promising seed cells for ischemia-related tissue engineering.

Researchers usually use oxygen, glucose, and serum deprivation (OGD) conditions to simulate the in vivo ischemic microenvironment, and extensive studies have been conducted to enhance the adaptation of MSCs to the ischemic microenvironment. OGD conditions were shown to induce apoptosis in BMSCs, while exendin-4 [119] and icariin [120] could protect BMSCs from OGD-induced apoptosis by attenuating the ER stress signaling pathway. The viability and adipogenic differentiation of AD-MSCs were also shown to be inhibited by OGD conditions, while supplementation with exogenous transforming growth factor- (TGF-) *β*3 [121] or AMPK [122] could protect AD-MSC survival and adipogenesis. Tian et al. [123] conducted the first experiment to simulate the effects of complex OGD conditions on NP-MSCs. They found that hypoxia combined with nutrient deprivation inhibited the proliferation and induced apoptosis of NP-MSCs, enhanced the activity of caspase 3, and also inhibited the expression of functional genes (proteoglycan, collagen-I, and collagen-II) and stem cell-related genes (Oct4, Nanog, Jagged, and Notch1), and activation of phosphatidylinositol 3-kinase (PI3K) by insulin-like growth factor-1 (IGF-1) attenuated this effect [123]. These findings provide a feasible method for improving the therapeutic effect of MSC transplantation (Table 3).

### 3.3. pH

Since the energy metabolism of the IVD cells mainly relies on glycolysis, lactic acid is produced at a higher rate during the metabolic process, resulting in a healthy IVD tissue pH between 7.2 and 7.0. Matrix acidity drops sharply during IDD, with pH as low as 6.5 best representing severe IDD, although pH can even drop to 5.6 [20, 124]. Wuertz et al. [104, 124] cultured BMSCs under four different pH conditions representative of the standard conditions, healthy, mildly, or severely degenerated IVD (pH 7.4, pH 7.1, pH 6.8, and pH 6.5) for several days. They found that acidic conditions inhibited BMSC viability, proliferation, and expression of matrix proteins [104, 124]. MSC therapies for IDD offer the greatest promise when applied at early stages of degeneration with pH levels of 6.8 or above [124]. Subsequently, Liang et al. [105] and Li et al. [125] studied the effect of acidic pH on AD-MSCs and observed similar results; that is, acidic conditions inhibited the viability, proliferation, and expression of aggrecan, collagen-I, and collagen-II of AD-MSCs. These findings suggest that acidic pH may be an important detrimental factor for MSC-induced regeneration of degenerative IVDs. Considering that NP-MSCs showed higher tolerance than AD-MSCs in hypoxic environment [44], Han et al. [126] compared the effect of IVD acidic environment on NP-MSCs and AD-MSCs. The results showed that NP-MSCs were less inhibited in terms of biological activity than AD-MSCs and thus may be more suitable for surviving in an acidic environment [126]. To determine the optimal timing of interventions that provide the greatest regenerative potential, Naqvi and Buckley [127] found a threshold at pH 6.8 below which BMSCs could not survive, proliferate, and accumulate NP-like matrix components (sGAG and collagen). This study suggested stratified targeting to identify suitable candidates through measurement of the local pH, thereby maximizing the efficacy for IVD cellular regenerative interventions [127].

With the deepening of research, the relevant mechanisms of the effect of acidic environment on the properties of MSCs in degenerative IVD have been gradually explored. Acid-sensing ion channels (ASICs) are a sort of voltage-insensitive Na^+^ channels that can be activated by extracellular H^+^ and are closely related to the acidic environment in degenerative discs [128, 129]. ASICs are widely distributed in various tissues of mammals, mainly including seven subtypes of ASIC1a, ASIC1b, ASIC1b_2_, ASIC2a, ASIC2b, ASIC3, and ASIC4. Activated ASICs predominantly conduct Na^+^ or K^+^, while homomeric ASIC1a is also Ca^2+^-permeable substantially [129, 130]. ASIC1a is expressed in BMSCs [131]. Cai et al. [132] found that acid-induced elevation of Ca^2+^ in BMSCs resulted in the activation of calpain and calcineurin, which in turn led to increased mitochondrial permeability and mitochondrial-mediated apoptosis. This study demonstrated that the acidic microenvironment of degenerating IVD can induce apoptosis of BMSCs by activating Ca^2+^-permeable ASIC1a [132]. Recently, a team [133] found that TGF-*β*3 can be used to prime BMSCs to maintain cell survival in an acidic environment and promote the accumulation of NP-like matrix, thereby assisting BMSCs to overcome the harsh microenvironment of IVDs. In addition to BMSCs, other groups have also conducted similar studies on NP-MSCs. The researchers found that acidic conditions can reduce the viability, proliferation, ECM synthesis, and stem cell-related gene expression of NP-MSCs but increase the expression of ASIC1 and ASIC3 [40, 134]. Sa12b, a wasp peptide, was found to reduce Ca^2+^ influx by inhibiting ASICs and Notch signaling pathways, thereby enhancing the biological activity of NP-MSCs in severely acidic environments [135]. Sa12b showed significant therapeutic potential in delaying IDD and improving LBP, providing a new perspective for the biological treatment of IDD [135] (Table 4).

### 3.4. Osmotic Cell Pressure

The aggrecan of the ECM is negatively charged under physiological conditions, and these negative charges explain the hyperosmolarity of the intervertebral disc [136, 137]. The extracellular osmotic pressure of normal IVDs ranges from 43 to -496 mOsm/L, which is significantly higher than that of other tissues. When the IVD degenerates, aggrecan is gradually lost, and the extracellular osmotic pressure of the IVD gradually decreases, and in severe cases, it can drop to about 300 mOsm [138]. Wuertz et al. [104] and Liang et al. [105] found that IVD-like hyperosmolarity (485 mOsm) significantly inhibited the viability, proliferation, and expression of aggrecan and collagen-I of BMSCs and AD-MSCs compared with standard conditions (280 mOsm). Furthermore, under combined conditions, osmolality and pH determine the effect of glucose [104, 105]. Tao et al. [139] observed that IVD-like hyperosmolarity (400 mOsm) also had similar inhibitory effects on NP-MSC viability, proliferation, and ECM protein synthesis, although NP-MSCs showed a certain tolerance to hyperosmolarity (400 mOsm). A team has reported the effect of a wider range of cartilage tissue-specific osmolarity (400-600 mOsm) on ADSMCs. Increased osmolarity (400-600 mOsm) inhibited the viability, proliferation, and chondrogenic differentiation potential of AD-MSCs in a dose- and time-dependent manner compared to 300 mOsm and resulted in a spherical appearance of the cells [140]. In addition, the viability of AD-MSCs was strongly dependent on the type of culture, with AD-MSCs in monolayer culture being more tolerant to increased osmolarity compared to AD-MSCs in suspension, alginate-agarose hydrogel, and pellet cultures, thus emphasizing the importance of choosing relevant in vitro conditions according to the specifics of clinical application [140].

However, some researchers have observed different results regarding the effect of hyperosmolarity on the chondrogenic differentiation of MSCs. Caron et al. [141] reported the effect of hyperosmolarity using NaCl as an osmolyte on the proliferation and chondrogenic differentiation of two chondrogenic progenitor cells (ATDC5 and BMSCs). During chondrogenic differentiation of ATDC5 and BMSCs, hyperosmolarity of +100 mOsm increased the expression of chondrogenic markers (collagen-II, collagen-X, and aggrecan), while hyperosmolarity of +200 mOsm inhibited ATDC5 proliferation and chondrogenesis compared with control levels (285-310 mOsm) [141]. This result suggested that the optimal osmolarity for chondrogenic differentiation of ATDC5 and BMSCs was about 400 mOsm. In addition, Caron et al. [141] pointed out that the nuclear factor of activated T-cell 5 (Nfat5, also known as TonEBP) is involved in regulating the expression of chondrogenic genes in progenitor cells under normal and hypertonic conditions by transcriptionally affecting the early expression of sox-9 (SRY- (sex-determining region Y-) box 9). Similar results were also observed in related studies of AD-MSCs. Ahmadyan et al. [142] assessed the expression of chondrogenic and hypertrophic markers and vascular endothelial growth factor (VEGF) secretion in AD-MSCs at three osmolarity levels using three different osmolytes, NaCl, sorbitol, and polyethylene glycol (PEG). As expected, all hyperosmolar conditions led to enhanced chondrogenesis with slightly varying degrees, and hypertonic conditions positively correlated with early expression of specific chondrocyte markers [142]. Nfat5 was observed to be involved in osmoadaptation of all treatments in varying degrees [142]. PEG and sorbitol have higher cartilage-promoting and hypertrophy-inhibiting effects compared to NaCl, which exacerbates hypertrophy [142, 143]. In this study, the inhibitory effect on AD-MSC proliferation was only observed in NaCl 550 mOsm, and the other treatments showed no inhibitory effect on cell proliferation [142]. These findings are basically consistent with the findings of Caron et al. [141].

Although it has been widely reported that hyperosmolarity promotes chondrogenic differentiation of MSCs, unlike Ahmadyan et al. [142] who reported that hyperosmolarity was positively correlated with the early expression of specific chondrocyte markers, the researchers found that higher or lower osmolarity may negatively affect chondrogenic differentiation. Zhang et al. [144] observed that high osmolarity (400 mOsm and 500 mOsm) inhibited the viability and proliferation of AD-MSCs compared with 300 mOsm, which was the same as the previous study. Interestingly, a slight increase in osmolarity to near physiological levels (400 mOsm) induced AD-MSCs to differentiate into an NP-like phenotype. Lower (300 mOsm) or higher (500 mOsm) osmolarity was found to decrease the expression of NP-like markers and ECM synthesis, suggesting a potential optimal osmotic window for successful differentiation of AD-MSCs [144]. In addition, it was reported for the first time that histone demethylase KDM4B induced NP-like differentiation of AD-MSCs through H3K9me3/2 targeting Foxa1/2 under hypertonic conditions [144]. Some researchers have observed that 480 mOsm hyperosmolarity can improve advanced cartilage formation by upregulating the expression of cartilage-specific markers and reducing mineralization rate and angiogenic potential [145]. Li et al. [146] cultured NP-MSCs in media at 300, 400, 430, and 500 mOsm, mimicking the osmotic pressures of serious degenerative, moderately degenerative, and healthy IVD. The results showed that, compared to 300 mOsm, hyperosmolarity of healthy IVD (430 and 500 mOsm) inhibited the proliferation and chondrogenic differentiation of NP-MSCs. The relative hypoosmotic condition of moderately degenerative IVD (400 mOsm) led to great proliferation and chondrogenic differentiation capacity [146]. These results suggest that the relatively hypotonic condition prevalent in degenerative IVD may provide a more permissive environment for chondrogenic differentiation of MSCs (Table 5).

### 3.5. Mechanical Stress

In daily life, the IVD tissue is stimulated by various mechanical loads such as compression, flexion and extension, hydrostatic pressure, torsion, and shear forces. Different mechanical loading intensities, durations, frequencies, and directions significantly affect the metabolism of IVD cells and ECM [147]. Static loading is thought to inhibit nutrient transport and necessary gas exchange and is associated with increased disc cell death and ECM catabolism, whereas dynamic loading is thought to promote macromolecular nutrient transport and is associated with increased ECM anabolism [148, 149]. These mechanical loads have an effect not only on the disc tissue but also on the biological behavior of the implanted MSCs. Previous studies have demonstrated that cyclic compressive loading can promote chondrogenic differentiation of BMSCs by upregulating TGF-*β* gene expression and protein synthesis [150–153]. These findings suggest that cyclic mechanical loading can be exploited to promote chondrogenic differentiation of the implanted BMSCs. Later, researchers conducted more in-depth studies to optimize the amplitude of cyclic compression that stimulates ECM synthesis. They found that low-amplitude dynamic compressive loading (5% amplitude) promoted ECM synthesis by inhibiting the transient receptor potential vanilloid 4 (TRPV4) without affecting the viability of AD-MSCs, whereas high-amplitude dynamic compressive loading inhibited their viability and ECM synthesis compared with the no-compression loading group [154]. Cyclic compression loading was also found to stimulate the proliferation and chondrogenic differentiation of AD-MSCs, and dynamic compression combined with exogenous sox-9 [155] showed additive effects on the chondrogenic differentiation of ADMCs. Although dynamic compressive loading promoted the proliferation and chondrogenic differentiation of MSCs, static compressive loading negatively affected the biological behavior of MSCs. Liang et al. [156] studied the effects of static compressive loading on the biological behavior of NP-MSCs. They found that static compressive loading significantly inhibited the viability, migration, colony-forming ability, and multiple differentiation potentials of NP-MSCs and reduced the stemness of NP-MSCs, which may be one of the mechanisms for the failure of endogenous repair of IDD [156].

Since the IVD tissue is usually not affected by a single type of mechanical load under physiological conditions, in order to study the effect of mechanical load on complex motion, some researchers established a fibrin-polyurethane scaffold compound culture system to analyze the effect of cyclic compressive load combined with surface shear stress on BMSCs [157, 158]. They found that the chondrogenic differentiation of BMSCs was affected by the frequency and amplitude of dynamic compression and surface shear stress, and the chondrogenic differentiation potential of BMSCs could be further enhanced by changing the frequency and compression amplitude [157, 158]. Similar to cyclic compressive loading, cyclic hydrostatic pressure was also shown to promote chondrogenic differentiation of BMSCs [159, 160] and AD-MSCs [161] in a dose- and time-dependent manner. Dai et al. [162] found that dynamic compression of dynamic hydrostatic pressure not only promoted the proliferation and differentiation of AD-MSCs into NP-like manifestations but also showed a cumulative effect on NP-like differentiation in combination with coculture with nucleus pulposus cells. To determine whether hydrostatic pressure and platelet-rich fibrin activate canonical or noncanonical Wnt signaling in BMSCs, BMSCs cocultured with platelet-rich fibrin (PRF) were hydrostatically loaded. [163] conducted an experiment to explore the biosignaling mechanism of BMSCs stimulated by hydrostatic pressure. They found that 120 kPa hydrostatic pressure activates both Wnt/*β*-catenin signaling and Wnt/Ca^2+^ signaling, and growth factors released by PRF may reverse the stress-promoting effect of Wnt/Ca^2+^ signaling [163]. This result indicated that Wnt signaling was involved in stress-promoting chondrogenesis in BMSCs cocultured with PRF, and the canonical and noncanonical pathways played different roles in this process. Some researchers have observed that intermittent compression of excessive hydrostatic pressure (1.0 MPa) induces apoptosis of NP-MSCs, and hypoxia can alleviate this negative effect [92].

In addition to compressive loads, shear forces, and hydrostatic pressure, the intervertebral disc tissue is also subjected to tensile loads, which also affect the biological behavior of the implanted MSCs. Connelly et al. [164] studied the effect of cyclic tensile loading (10%, 1 Hz) on the chondrogenic differentiation of BMSCs and found that cyclic tensile loading specifically stimulated the synthesis of collagen-I but had no effect on the synthesis of collagen-II, aggrecan, or osteocalcin. Overall, this study demonstrated that cyclic tensile loading promoted the fibrochondrocyte-like differentiation of BMSCs, and Baker et al. [165] observed similar results in a subsequent study. Recently, Abusharkh et al. [166] found that coculture of AD-MSCs with articular chondrocytes (AChs) under cyclic tensile stress of 10% and 1 Hz could promote the production of cartilage ECM of AD-MSCs, and 1 : 3 AChs : AD-MSCs was a good coculture ratio. The results of this study provide new ideas for optimizing tissue engineering for stem cell therapy (Table 6).

### 3.6. Inflammatory Cytokines and Proteinases

IDD is a complex process. Activation of autoimmune responses is an important pathological mechanism leading to IDD, and inflammatory mediators may be a key factor in its occurrence and progression. Under physiological conditions, the NP tissue is trapped by the surrounding AF and CEPs, and this unique structure isolates the NP from the immune system of the host, thereby maintaining the IVD immune privilege [167, 168]. When IDD occurs, the AF is damaged to produce fissures, and the exposed NP tissue activates the autoimmune response system, thereby stimulating immunocyte activation and infiltration of inflammatory mediators. Meanwhile, the recruitment of immunocytes and the infiltration of inflammatory mediators in turn accelerate the progression of IDD [169, 170]. The autoimmune response to NP involves various types of immunocytes, including macrophages, T lymphocytes, and neutrophils, and macrophages may play a significant role in this pathological process [169]. In IDD patients, the expression levels of various proinflammatory cytokines are upregulated. Common highly expressed proinflammatory cytokines include tumor necrosis factor-*α* (TNF-*α*), interleukin-1*α*/*β* (IL-1*α*/*β*), IL-6, IL-17, IL-8, interferon-*γ* (IFN-*γ*), prostaglandin E2 (PGE2), and substance P, among which TNF-*α*, IL-1*β*, IL-6, and IL-17 are the key inflammatory mediators [171–173]. By activating the nuclear factor kappa-B (NF-*κ*B), mitogen-activated protein kinase (MAPK), and Toll-like receptor (TLR) signaling pathways, these proinflammatory cytokines can induce disc degeneration by promoting catabolic enzymes, such as a disintegrin and metalloproteinase with thrombospondin motif- (ADAMTS-) 4 and -5 and MMP-1, -2, -3, -4, -13, and -14, and decreasing anabolic ECM proteins, such as aggrecan and collagen-II [174, 175]. In addition to proinflammatory cytokines, anti-inflammatory cytokines, such as TGF-*β*, growth, and differentiation factor 5 (GDF-5), GDF-6, IL-4, and IL-10, can also be observed in IDD patients, which can promote IVD repair and relieve pain symptoms to a certain extent [173, 176]. The paradoxical effects of the expression of these inflammatory mediators are closely related to macrophage polarization and the distribution of different types of macrophages [177, 178].

The expression of inflammatory mediators can not only affect the degeneration process of the IVD but also affect the biological behavior of the implanted MSCs. Felka et al. [77] found that supplementation of IL-1*β* had a negative effect on the chondrogenic differentiation potential of MSCs, while hypoxia reduced the inhibitory effect of IL-1*β* on the chondrogenic differentiation of BMSCs. Subsequently, some researchers found that IL-1*β* could induce BMSC migration, adhesion, and leukocyte chemotaxis by activating the NF-*κ*B signaling pathway but did not affect the viability and proliferation of BMSCs [179, 180]. TNF-*α* has also been observed to enhance the migratory capacity of BMSCs through the NF-*κ*B signaling pathway [181–183]. Interestingly, similar results were not observed for all types of MSCs. Brandt et al. [184] studied the effect of different inflammatory conditions on the functional properties of AD-MSCs. They found that high concentrations of proinflammatory cytokines (IL-1*β*: 10 ng/mL and/or TNF-*α*: 50 ng/mL) promoted the proliferation and osteogenic differentiation of AD-MSCs but decreased cell viability and chondrogenic and adipogenic differentiation potential. In contrast, Mohammadpour et al. [185] pointed out that TNF-*α* alone had no effect on the proliferation of AD-MSCs, but combined with IFN-*γ* could significantly promote the proliferation of AD-MSCs. Cheng et al. [186] investigated the regulatory effects of TNF-*α* at high and low concentrations on the biological behaviors of healthy rat NP-MSCs. It was found that a high concentration of TNF-*α* (50-200 ng/mL) could induce apoptosis of NP-MSCs, whereas a relatively low TNF-*α* concentration (0.1-10 ng/mL) promoted the proliferation and migration of NP-MSCs but inhibited their differentiation toward NP-like cells [186]. Moreover, the NF-*κ*B signaling pathway was activated during the differentiation of NP-MSCs inhibited by TNF-*α* [186]. Another team [187] exposed UC-MSCs to a proinflammatory cytokine environment in vitro and observed that IL-1*β* and TNF-*α*, although inhibited the proliferation and adipogenesis of UC-MSCs, enhanced their chondrogenic differentiation capacity. The differences in these findings may be related to the different types of MSCs, culture conditions, and concentrations of proinflammatory cytokines.

As one of the important proinflammatory cytokines in IDD, the effects of IL-6 and IL-17 on the biological behavior of MSCs have also been investigated. Studies have shown that IL-6 inhibits the chondrogenic differentiation of BMSCs, and the JAK/STAT pathway is activated during this process [187, 188]. In addition, IL-6 was also found to limit BMSC proliferation but improve their migration [189]. IL-17 is a proinflammatory cytokine mainly secreted by Th17 cells. Huang et al. [190] reported that IL-17 could directly stimulate BMSC proliferation, migration, and osteoblast differentiation, and these response mechanisms were shown to involve the production of reactive oxygen species. Mojsilović et al. [191] also observed the promoting effect of IL-17 on the proliferation of BMSCs and pointed out that mitogen-activated protein kinase (MAPK) signaling pathways mediated by p38 and extracellular signal-regulated kinase (ERK) were involved in this process. Recently, a team found that IL-17 pretreatment can be used to enhance the homing ability and immunosuppressive function of BMSCs, thereby achieving the purpose of prolonging graft survival and reducing graft rejection. This finding provides a novel and useful research focus for developing MSCs for transplantation techniques [192].

Not only proinflammatory cytokines but also anti-inflammatory cytokines in IDD can regulate the biological behaviors of engrafted MSCs. In mammals, the TGF-*β* family includes three TGF-*β* members, TGF-*β*1, TGF-*β*2, and TGF-*β*3. TGF-*β* signaling plays a critical role in IVD development, growth, and tissue homeostasis, but overactivation of TGF-*β* signaling can lead to aggravation of IDD [193]. GDF-5 and GDF-6 are members of the TGF-*β* superfamily, which also play an important role in spine development. Studies have shown that TGF-*β*, GDF-5, and GDF-6 can all promote the differentiation of MSCs to an NP-like phenotype [80, 194–197]. And compared with TGF-*β* or GDF-5, GDF-6 can drive MSCs to produce more proteoglycan-rich ECM, which is more suitable for promoting the differentiation of MSCs to NP-like phenotype [196]. Furthermore, the effect is more pronounced in AD-MSCs than BMSCs [196]. Later, Li et al. [198] found that TGF-*β*3 mainly promotes the ingrowth of AD-MSCs into cartilage through the Wnt5a/*β*-catenin signaling pathway, which is expected to provide new ideas for the field of cartilage regeneration. IL-4 is another important anti-inflammatory cytokine in the body. Pretreatment with IL-4 can improve the migration ability of MSCs and the expression level of certain proteins, which has a beneficial effect on the regeneration process of MSCs [189, 199, 200].

In addition to common types of MSCs, researchers have also explored the effects of inflammatory cytokines on rare types of MSCs. Li et al. [201] found that proinflammatory cytokines (IL-1, IL-6, and IL-8) promoted the proliferation of PMSCs in a dose-dependent manner, peaking at concentrations of 10 ng/mL of IL-1 and IL-6 and 150 ng/mL of IL-8. In addition, anti-inflammatory cytokine (IL-4) inhibited the proliferation of PMSCs in a dose-dependent manner [201]. However, Zhang et al. [202] separately studied the interaction between IL-1*β* and PMSCs, and the results showed that IL-1*β* (20 ng/mL or 30 ng/mL) regulates programmed death ligand 1 (PD-L1) expression through JAK and NF-*κ*B pathways, thereby inhibiting the adhesion and proliferation of PMSCs. IFN-*γ* was also found to inhibit the proliferation and migration of PMSCs [203]. Recently, a team [204] conducted a similar study using PB-MSCs, and they observed that compared with the unstimulated control group, IL-1*β* promoted cell migration, while TNF-*α* inhibited the migration of PB-MSCs. In order to compare the different responses of various types of MSCs to inflammatory cytokines, comparative studies between stem cells have also been carried out. Park et al. [205] evaluated the effect of anti-inflammatory cytokines (TGF-*β*3) on the differentiation of BMSCs, AD-MSCs, and AF-MSCs. Compared with the control group without TGF-*β*3, the chondrogenic differentiation potential of these three types of MSCs have been improved. Furthermore, the chondrogenic potential of AF-MSCs were relatively low compared to BMSCs and AD-MSCs [205]. In a previous study, Calle et al. [206] compared the effects of proinflammatory cytokines on the migration properties of MSCs and showed that IL-1*β* promoted the migration of AD-MSCs and PB-MSCs compared with the control group. Borem et al. [207] cultured human AD-MSCs and AMSCs in medium supplemented with IL-1*β* and TNF-*α* to directly compare the impact of IDD inflammation on their effector functions. Compared with noninflammatory controls, inflammatory cultures promoted the proliferation of MSCs but inhibited their chondrogenic differentiation, and these effects were more pronounced in AD-MSCs [207]. These findings may help researchers understand which source of MSCs may be the best choice for IVD regeneration.

MMPs are a family of neo-dependent proteolytic enzymes that play an important role in the catabolism of ECM. Their activity is modulated by specific inhibitors known as tissue inhibitors of metalloproteinases (TIMPs). Dysregulated expression and activity of MMPs is an important cause of ECM catabolism in IDD [208]. Increased expression of MMPs, including MMP-1, -2, -3, -7, -8, -9, -10, -13, and -14 and ADAMTS-4, has been repeatedly observed during IDD, some of which are related with the severity of degradation [21, 209]. MMPs can not only regulate the matrix metabolic activities of IVD tissues but also regulate the biological behavior of the implanted MSCs. Studies have found that membrane-type MMP-1 (MT1-MMP) promotes the proliferation and migration of BMSCs through the Wnt signaling pathway [210–213] and induces the differentiation of BMSCs into NP-like cells [212]. In addition, silencing MMP-2 also inhibited the proliferation and migration of MSCs [210, 214]. MMPs also affect the biological behavior of other types of MSCs. MMP-9 can promote the proliferation and migration of AD-MSCs [215]. Overexpression of MMP-3 decreased the expression level of collagen-I in AD-MSCs [216], and its downregulation promoted the chondrogenic differentiation of NP-MSCs [217]. The increased expression of MMP-2 can promote the proliferation and migration of UC-MSCs while maintaining their chondrogenic differentiation potential [218]. There are many types of MMPs, which play an important role in regulating the biological behavior of the implanted stem cells. However, there are still relatively few related studies, so it is necessary to carry out extensive related research in the follow-up (Table 7).

## 4. Conclusions

Although the biological behavior of the implanted MSCs will be affected by various IVD microenvironmental factors, they can also secrete some bioactive factors through the paracrine pathway to regulate the microenvironment in which they are located, so as to better adapt to the IVD microenvironment. With the deepening of the understanding of microenvironmental factors, more and more researchers have begun to use microenvironmental pretreatment or develop various scaffold materials to reduce the adverse effects of the microenvironment on implanted MSCs, so as to maximize the therapeutic effect of stem cells for repairing degenerated IVDs. The effect of IVD microenvironment on the biological behavior of MSCs is a complex process with multiple factors. Although the current understanding of it has greatly improved, the interaction of transplanted MSCs with the complex microenvironment of IVD and the exact mechanisms behind it are still not fully understood. In addition, stem cell therapy for IDD is still in the clinical trial stage, and there is still some distance from the final clinical application. For these reasons, we need to continue to conduct more in-depth research. Only by fully understanding these fields can we develop corresponding tissue engineering techniques to help MSCs better survive, proliferate, and synthesize ECM proteins in IVD tissues, so that stem cells can play a greater role in regeneration and repair in the treatment of IDD.

## Figures and Tables

**Table 1:** Common types of MSCs and their characteristics.

Cell sources	Advantages	Disadvantages
BMSCs	High chondrogenic differentiation potential	Cumbersome and invasive procedure to obtain and low cell density in bone marrow aspirate
AD-MSCs	Ease of bulk availability, low donor site morbidity, and high proliferation rates	Lower differentiation potential than BMSCs
UC-MSCs	Easy to obtain, low immunogenicity, and no ethical barriers	Insufficient differentiation potential and high treatment costs
NP-MSCs	Well adapted to the microenvironment of the IVD	Lack of standard, reliable and efficient separation, and purification methods

AD-MSCs: adipose-derived mesenchymal stem cells; BMSCs: bone marrow-derived mesenchymal stem cells; IVD: intervertebral disc; NP-MSCs: nucleus pulposus-derived mesenchymal stem cells; UC-MSCs: umbilical cord-derived mesenchymal stem cells.

**Table 2:** Effects of hypoxia on the biological behavior of MSCs.

Cell sources	Year	Team	Journal	Results	Reference
BMSCs	2004	Risbud et al.	Spine	Hypoxia (2% O_2_) and TGF-*β*1 induced the differentiation of BMSCs to a NP-like phenotype.	[78]
	2006	Grayson et al.	J Cell Physiol	Hypoxia (2% O_2_) enhanced BMSC viability, proliferation, and expression of stemness genes (Oct4 and Rex-1) compared with normoxia.	[72]
	2007	Grayson et al.	Biochem Biophys Res Commun	Hypoxia (2% O_2_) promoted the proliferation of BMSCs and maintained their multilineage capabilities compared with normoxia.	[73]
	2008	Kanichai et al.	J Cell Physiol	Hypoxia (2% O_2_) promoted the chondrogenic differentiation of BMSCs compared with normoxia.	[81]
	2009	Felka et al.	Osteoarthritis Cartilage	Hypoxia (2% O_2_) reduced the negative effect of IL-1*β* on chondrogenic differentiation of BMSCs.	[77]
	2011	Tsai et al.	Blood	Hypoxia (1% O_2_) promoted BMSC proliferation and maintained their chondrogenic differentiation potential compared with normoxia.	[74]
	2011	Müller et al.	Cell Transplant	Hypoxia (4% O_2_) promoted chondrogenic differentiation of BMSCs compared with normoxia.	[79]
	2011	Stoyanov et al.	Eur Cell Mater	Hypoxia (2% O_2_) and GDF5 (100 ng/mL) promoted the differentiation of BMSCs to a NP-like phenotype.	[80]
	2011	Raheja et al.	Cell Biol Int	Hypoxia (1% O_2_) inhibited BMSC migration compared with normoxia.	[76]
	2018	Wang et al.	Stem Cells Int	Hypoxic preconditioning (CoCl_2_, 100 *μ*M, 24 h) enhanced BMSC viability, migration, and expression of aggrecan and collagen-II but inhibited their proliferation.	[75]
	2021	Peck et al.	Cartilage	Hypoxic preconditioning (2% O_2_) promoted BMSC viability and ECM production.	[82]

AD-MSCs	2012	Chung et al.	Res Vet Sci	Hypoxia (1% and 5% O_2_) inhibited the proliferation of AD-MSCs and BMSCs compared with normoxia, and AD-MSCs exhibited higher proliferative potential than BMSCs.	[91]
	2013	Portron et al.	PLoS One	Hypoxic preconditioning (5% O_2_) enhanced the chondrogenic differentiation of AD-MSCs in vitro but not in vivo.	[84]
	2014	Choi et al.	Journal of Asian Scientific Research	Hypoxia (2% O_2_) promoted AD-MSC viability and proliferation compared with normoxia.	[86]
	2015	Fotia et al.	Cytotechnology	Hypoxia (1% O_2_) promotes AD-MSC proliferation and expression of stemness genes (Nanog and Sox-2) compared with normoxia.	[87]
	2018	Takahashi et al.	Cell Transplant	Hypoxia (1% O_2_) inhibited AD-MSC viability but promoted their proliferation compared with normoxia.	[89]
	2019	Deng et al.	J Cell Physiol	Hypoxia (5% O_2_) promoted AD-MSC proliferation and chondrogenic differentiation potential compared with normoxia.	[90]
	2020	Hwang et al.	Tissue Eng Regen Med	Hypoxic preconditioning (1% O_2_) promoted AD-MSC proliferation, migration, and chondrogenic differentiation.	[83]
	2021	Govoni et al.	Adv Med Sci	Severe hypoxic preconditioning (0.5% O_2_) promoted early chondrogenesis in AD-MSCs.	[85]

NP-MSCs	2013	Li et al.	Cells Tissues Organs	Hypoxia (2% O_2_) inhibited the viability and proliferation of AD-MSCs and NP-MSCs but promoted their chondrogenic differentiation, and NPMSCs exhibited higher biological activity than AD-MSCs.	[44]
	2021	He et al.	Autophagy	Hypoxia (CoCl_2_) alleviated hydrostatic pressure-induced NP-MSC apoptosis.	[92]

UC-MSCs	2015	Lee et al.	Stem Cells	Hypoxia (2.2% O_2_) promoted UC-MSC proliferation and migration compared with normoxia.	[93]

PMSCs	2012	Yang et al.	The Spine Journal	Hypoxia (5% O_2_) promoted the early proliferation and differentiation of PMSCs into NP-like cells of compared with normoxia.	[94]
	2014	Ni et al.	The Spine Journal	Hypoxia (5% O_2_) promoted the proliferation and differentiation of PMSCs into NP-like cells of compared with normoxia.	[95]
	2016	Choi et al.	J Cell Biochem	Hypoxia (1% O_2_) promoted PMSC migration compared with normoxia.	[96]
	2017	Li et al.	The Journal of thoracic and cardiovascular surgery	Hypoxia (CoCl_2_) promoted PMSC proliferation.	[97]

AF-MSCs	2017	Kwon et al.	Tissue Eng Regen Med	Hypoxia (5% O_2_) promoted AF-MSC proliferation and stemness maintenance compared with normoxia.	[98]
	2020	Casciaro et al.	Mech Ageing Dev	Hypoxia (1% O_2_) promoted AF-MSC proliferation and stemness maintenance compared with normoxia.	[99]

SMSCs	2020	Silva et al.	Glycoconj J	Hypoxia (5% O_2_) promoted chondrogenic differentiation of SMSCs compared with normoxia.	[101]

PB-MSCs	2022	Wang et al.	Front Endocrinol	Hypoxia (5% O_2_) enhanced the proliferation, stemness, and multidirectional differentiation potential of PBSMCs compared with normoxia	[100]

AD-MSCs: adipose-derived mesenchymal stem cells; AF-MSCs: amniotic fluid-derived mesenchymal stem cells; BMSCs: bone marrow-derived mesenchymal stem cells; ECM: extracellular matrix; IL-1*β*: interleukin-1*β*; IVD: intervertebral disc; NP: nucleus pulposus; NP-MSCs: nucleus pulposus-derived mesenchymal stem cells; PB-MSCs: peripheral blood-derived mesenchymal stem cells; PMSCs: placenta-derived mesenchymal stem cells; SMSCs: synovial-derived mesenchymal stem cells; TGF: transforming growth factor; UC-MSCs: umbilical cord-derived mesenchymal stem cells.

**Table 3:** Effects of nutrient deficiency on the biological behavior of MSCs.

Cell sources	Year	Team	Journal	Results	Reference
BMSCs	2007	Potier et al.	Tissue Eng	Serum deprivation (1% and 0% FBS) combined with hypoxia inhibited BMSC viability.	[112]
	2008	Wuertz et al.	Spine	IVD-like glucose (1.0 mg/mL) promoted BMSC proliferation and expression of aggrecan and collagen-I but had no effect on their viability.	[104]
	2015	Farrell et al.	Osteoarthritis Cartilage	Glucose deprivation (1.0 g/L) inhibited BMSC viability.	[107]
	2015	Naqvi and Buckley	J Anat	Under hypoxic conditions (5% O_2_), IVD-like glucose (5 mM) promoted the accumulation of sGAG and collagen compared with standard conditions (25 mM), whereas low glucose (1 mM) inhibited BMSC viability, proliferation, and accumulation of sGAG and collagen.	[108]
	2016	He et al.	Int J Mol Med	Oxygen, glucose, and serum deprivation inhibited BMSC viability.	[119]
	2020	Liu et al.	Life Sci	Oxygen, glucose, and serum deprivation inhibited BMSC viability and proliferation.	[120]

AD-MSCs	2007	Parker et al.	Cytotherapy	Both low-serum (0.5%) and serum-free maintained the chondrogenic differentiation ability of AD-MSCs.	[110]
	2012	Liang et al.	J Transl Med	IVD-like glucose (1.0 mg/mL) slightly inhibited AD-MSC viability but increased aggrecan expression.	[105]
	2016	Safwani et al.	Cytotechnology	Serum-free inhibited AD-MSC viability and proliferation but promoted their chondrogenic differentiation.	[111]
	2018	Takahashi et al.	Cell Transplant	Serum-free conditions inhibited the viability of AD-MSCs and BMSCs, and AD-MSCs exhibited a higher survival rate than BMSCs.	[89]
	2018	Ghorbani et al.	Nat Prod Res	Glucose-serum deprivation inhibited AD-MSC viability.	[114]
	2018	Wu et al.	Exp Ther Med	Oxygen, glucose, and serum deprivation inhibited AD-MSC viability.	[121]
	2018	Li et al.	Biochim Biophys Acta Mol Cell Biol Lipids	Oxygen, glucose, and serum deprivation inhibited AD-MSC viability.	[122]
	2020	Abdolmaleki et al.	Cell Tissue Bank	Glucose-serum deprivation inhibited AD-MSC viability and proliferation.	[115]
	2021	Pang et al.	Drug Des Devel Ther	Serum deprivation combined with hypoxia inhibited AD-MSC viability.	[113]
	2022	Pan et al.	Cytotechnology	Serum deprivation inhibited AD-MSC viability, proliferation, and expression of stemness genes (Oct4, Nanog, and Sox-2).	[116]
	2022	Yang et al.	Biosci Rep	Glucose deprivation combined with hypoxia (1% O_2_) inhibited AD-MSC viability, proliferation, and migration.	[109]

NP-MSCs	2009	Jünger et al.	Spine	Limited glucose (2.0 mg/mL) inhibited NP-MSC viability compared with sufficient glucose (4.5 mg/mL).	[106]
	2020	Tian et al.	J Orthop Surg Res	Oxygen, glucose, and serum deprivation inhibited NP-MSC viability, proliferation, and expression of aggrecan, collagen-I, and collagen-II.	[123]

PMSCs	2009	Huang et al.	Stem Cell Rev and Rep	Hypoxia (1% O_2_) and serum deprivation did not induce apoptosis in PMSCs.	[117]
	2010	Huang et al.	Cell biology international	Hypoxia (1% O2) promoted the proliferation of PMSCs, whereas serum deprivation inhibited the growth of PMSCs.	[118]

AD-MSCs: adipose-derived mesenchymal stem cells; BMSCs: bone marrow-derived mesenchymal stem cells; FBS: fetal bovine serum; IVD: intervertebral disc; NP-MSCs: nucleus pulposus-derived mesenchymal stem cells; PMSCs: placenta-derived mesenchymal stem cells; sGAG: sulphated glycosaminoglycans.

**Table 4:** Effects of pH on the biological behavior of MSCs.

Cell sources	Year	Team	Journal	Results	Reference
BMSCs	2008	Wuertz et al.	Spine	IVD-like acidity (pH 6.8) inhibited BMSC viability, proliferation, and expression of aggrecan and collagen-I.	[104]
	2009	Wuertz et al.	Biochem Biophys Res Commun	Increased acidity inhibited BMSC viability, proliferation, and expression of aggrecan and collagen-I.	[124]
	2016	Naqvi and Buckley	Spine	Acidity with pH below 6.8 inhibited BMSC viability, proliferation, and accumulation of sGAG and collagen.	[127]
	2019	Cai et al.	Biosci Rep	Degenerated IVD-like acidity inhibited BMSC viability and proliferation.	[132]
	2021	Gansau et al.	Eur Cell Mater	Low pH (pH 6.8 and pH 6.5) inhibited AD-MSC viability and accumulation of sGAG and collagen compared with standard pH (pH 7.4).	[133]

AD-MSCs	2012	Laing et al.	J Transl Med	IVD-like acidity (pH 6.8) inhibited AD-MSC viability, proliferation, and expression of aggrecan and collagen-I.	[105]
	2012	Li et al.	Exp Biol Med	Acidic pH inhibited AD-MSC viability, proliferation, and expression of aggrecan, collagen-I and collagen-II.	[125]
	2014	Han et al.	Cells Tissues Organs	Acidic pH inhibited AD-MSC and NP-MSC viability, proliferation, and expression of aggrecan, collagen-I, and collagen-II, and NP-MSCs exhibited higher tolerance to acidity than AD-MSCs.	[126]

NP-MSCs	2017	Liu et al.	Stem Cells Dev.	Acidic pH inhibited NP-MSC viability, proliferation, and ECM synthesis.	[40]
	2021	Ding et al.	Aging	Acidic pH (pH 6.6) inhibited NP-MSC proliferation and induced their senescence.	[134]
	2022	Wang et al.	Front Bioeng Biotechnol	Acidic conditions (pH 6.2) inhibited NP-MSC viability, proliferation, and ECM synthesis.	[135]

AD-MSCs: adipose-derived mesenchymal stem cells; BMSCs: bone marrow-derived mesenchymal stem cells; ECM: extracellular matrix; IVD: intervertebral disc; NP-MSCs: nucleus pulposus-derived mesenchymal stem cells; sGAG: sulphated glycosaminoglycans.

**Table 5:** Effects of osmotic cell pressure on the biological behavior of MSCs.

Cell sources	Year	Team	Journal	Results	Reference
BMSCs	2008	Wuertz et al.	Spine	IVD-like osmolarity (485 mOsm) inhibited BMSC viability, proliferation, and expression of aggrecan and collagen-I compared with controls (280 mOsm).	[104]
	2013	Caron et al.	Bone	The increased osmolarity (380 mOsm) promoted the chondrogenic differentiation of BMSCs compared with controls (285 ± 5 mOsm).	[141]

AD-MSCs	2012	Liang et al.	J Transl Med	IVD-like osmolarity (485 mOsm) inhibited AD-MSC viability, proliferation, and expression of aggrecan and collagen-I compared with controls (280 mOsm).	[105]
	2016	Potočar et al.	PLoS One.	The increased osmolarity (400-600 mOsm) inhibited AD-MSC viability, proliferation, and chondrogenic differentiation potential compared with controls (300 mOsm).	[140]
	2018	Ahmadyan et al.	Appl Biochem Biotechnol	All hyperosmolar conditions (350, 450, and 550 mOsm) promoted the chondrogenic differentiation of AD-MSCs, and only NaCl 550 mOsm inhibited AD-MSC proliferation.	[142]
	2018	Ahmadyan et al.	Cell Mol Biol	Hyperosmolar conditions (450 mOsm) promoted the chondrogenic differentiation of AD-MSCs compared with controls (350 mOsm).	[143]
	2020	Zhang et al.	Mol Cell Biochem	Compared with controls (300 mOsm), 400 mOsm osmolarity promoted NP-like differentiation of AD-MSCs, whereas 500 mOsm osmolarity inhibited NP-like differentiation of AD-MSCs and increased osmolarity (400 and 500 mOsm) inhibited their viability and proliferation.	[144]
	2021	Alinezhad-Bermi et al.	In Vitro Cell Dev Biol Anim	Certain dose of hyperosmolarity (480 mOsm) promoted the chondrogenic differentiation of AD-MSCs compared with controls (350 mOsm).	[145]

NP-MSCs	2013	Tao et al.	Cell Biol Int	IVD-like osmolarity (400 mOsm) slightly inhibited NP-MSC viability and significantly inhibited their proliferation and expression of sox-9, aggrecan, and collagen-II compared with controls (280 mOsm).	[139]
	2018	Li et al.	Cells Tissues Organs	Compared with controls (300 mOsm), the hyperosmolarity (430 and 500 mOsm) of healthy IVD inhibited the proliferation and chondrogenic differentiation of NP-MSCs, whereas the relative hyperosmolarity (400 mOsm) promoted their proliferation and chondrogenic differentiation.	[146]

AD-MSCs: adipose-derived mesenchymal stem cells; BMSCs: bone-marrow mesenchymal stem cells; IVD: intervertebral disc; NP: nucleus pulposus; NP-MSCs: nucleus pulposus-derived mesenchymal stem cells.

**Table 6:** Effects of mechanical stress on the biological behavior of MSCs.

Cell sources	Year	Team	Journal	Results	Reference
BMSCs	2003	Angele et al.	J Orthop Res	Cyclic hydrostatic pressure promoted chondrogenic differentiation of BMSCs.	[159]
	2004	Angele et al.	Biorheology	Cyclic compressive loading promoted chondrogenic differentiation of BMSCs.	[150]
	2004	Huang et al.	Stem Cells	Cyclic compressive loading (1 Hz, 10% magnitude) promoted chondrogenic differentiation of BMSCs	[152]
	2005	Huang et al.	Stem Cells	Cyclic compressive loading (1 Hz, 15% magnitude) promoted chondrogenic differentiation of BMSCs.	[151]
	2006	Miyanishi et al.	Tissue Eng	Different levels of cyclic hydrostatic pressure (1 Hz, 0.1, 1, and 10 MPa) differentially regulated BMSC chondrogenesis in the presence of TGF-*β*3.	[160]
	2007	Mouw et al.	Stem Cells	Cyclic compressive loading (1 Hz, 10% ± 3% magnitude) promoted the chondrogenic differentiation of BMSCs.	[153]
	2010	Li et al.	Tissue Eng Part A	Cyclic compressive loading combined with surface shear force promoted the chondrogenic differentiation of BMSCs.	[157]
	2010	Li et al.	J Cell Mol Med	Cyclic compressive loading combined with surface shear stress promoted the chondrogenic differentiation of BMSCs.	[158]
	2010	Connelly et al.	Tissue Eng Part A	Cyclic tensile loading (1 Hz, 10% magnitude) specifically promoted the synthesis of collagen-I by BMSCs.	[164]
	2011	Baker et al.	Tissue Eng Part A	Cyclic tensile loading (1 Hz, 3% magnitude) promoted ECM synthesis by BMSCs.	[165]
	2018	Gan et al.	Stem Cells Int	Compared with no-compression controls, low-magnitude dynamic compressive loading (5%, 1 Hz) promoted ECM synthesis without affecting BMSC viability, whereas high-magnitude dynamic compressive loading (10% and 20%, 1 Hz) inhibited their viability and ECM synthesis.	[154]
	2022	Cheng et al.	Tissue Eng Regen Med	Hydrostatic pressure promoted chondrogenic differentiation of BMSCs cocultured with RPF.	[163]

AD-MSCs	2009	Ogawa et al.	Tissue Eng Part A	Cyclic hydrostatic pressure (0.5 Hz, 0-0.5 MPa) promoted the chondrogenic differentiation of AD-MSCs.	[161]
	2014	Dai et al.	J Biomech	Dynamic compression of intermittent dynamic hydrostatic pressure promoted the proliferation of AD-MSCs and induces their differentiation into NP-like cells.	[162]
	2015	Zhang et al.	Eur Rev Med Pharmacol Sci	Cyclic compressive loading combined with exogenous sox-9 promoted the proliferation and chondrogenic differentiation of AD-MSCs.	[155]
	2021	Abusharkh et al.	In Vitro Cell Dev Biol Anim	High cyclic tensile loading (10% magnitude) promoted ECM synthesis without affecting the viability of AD-MSCs.	[166]

NP-MSCs	2018	Liang et al.	Stem Cells Int	Compressive loading (1 MPa) inhibited NP-MSC viability, migration, and expression of stemness genes (Sox-2 and Oct4).	[156]
	2021	He et al.	Autophagy	Hydrostatic pressure (1.0 MPa) inhibited NP-MSC viability.	[92]

AD-MSCs: adipose-derived mesenchymal stem cells; BMSCs: bone marrow-derived mesenchymal stem cells; ECM: extracellular matrix; TGF-*β*3: transforming growth factor-*β*3; NP: nucleus pulposus; NP-MSCs: nucleus pulposus-derived mesenchymal stem cells.

**Table 7:** Effects of inflammatory cytokines and proteases on the biological behavior of MSCs.

	Cell sources	Year	Team	Journal	Results	Reference
Inflammatory cytokines	BMSCs	2007	Ponte et al.	Stem Cells	TNF-*α* (1 ng/mL) promoted BMSC migration.	[181]
		2009	Felka et al.	Osteoarthritis Cartilage	IL-1*β* (2 ng/mL) inhibited the chondrogenic differentiation of BMSCs, while hypoxia (2% O_2_) reduced the inhibitory effect of IL-1*β* on the chondrogenic differentiation of BMSCs.	[77]
		2009	Huang et al.	Cell Death Differ	IL-17 (50 ng/mL) promoted BMSC proliferation and migration.	[190]
		2011	Mojsilović et al.	Cell Tissue Res	IL-17 (5-50 ng/mL) and bFGF (1 ng/mL) promoted the proliferation of BMSCs by activating p38 and ERK-mediated MAPK signaling pathway.	[191]
		2011	Gantenbein-Ritter et al.	Eur Spine J	TGF-*β*1 (10 ng/mL) and GDF-5 (100 ng/mL) promoted the differentiation of BMSCs into NP-like cells.	[194]
		2011	Stoyanov et al.	Eur Cell Mater	Hypoxia (2% O_2_) and GDF5 (100 ng/mL) promoted the differentiation of BMSCs to a NP-like phenotype.	[80]
		2017	Wang et al.	Cell Death Dis	TNF-*α* promoted (30 ng/mL) BMSC migration.	[182]
		2018	Teixeira et al.	Spine	IL-1*β* (10 ng/mL) promoted BMSC migration but had no effect on cell viability.	[180]
		2018	Ma et al.	Ann Transplant	IL-17 (50 ng/mL) increased the homing and immunosuppressive abilities of BMSCs.	[192]
		2019	Kasprzycka et al.	Stem Cell Res Ther	IL-4 (50 ng/mL) and IL-6 (50 ng/mL) inhibit BMSC proliferation but promote their migration.	[189]
		2021	Xie et al.	Nat Commun	High concentration of TNF-*α* (100 ng/mL) promoted the directional migration of BMSCs.	[183]
	AD-MSCs	2014	Clarke et al.	Arthritis Res Ther	Compared with TGF-*β* (10 ng/mL) or GDF-5 (100 ng/mL), GDF-6 (100 ng/mL) was more suitable for promoting the differentiation of MSCs to NP-like phenotype, and it was more pronounced in AD-MSCs than in BMSCs.	[196]
		2016	Colombier et al.	Stem Cells	TGF-*β*1 (10 ng/mL) combined with GDF-5 (100 ng/mL) promoted the chondrogenic differentiation of AD-MSCs.	[197]
		2016	Mohammadpour et al.	Immunopharmacology and Immunotoxicology	IFN-*γ* (10 ng/mL) alone or in combination with TNF-*α* (10 ng/mL) promoted the proliferation of AD-MSCs, while TNF-*α* (10 ng/mL) alone had no effect on the proliferation of AD-MSCs.	[185]
		2018	Brandt et al.	Int J Mol Sci.	High concentrations of proinflammatory cytokines (IL-1*β*: 10 ng/mL and/or TNF-*α*: 50 ng/mL) promoted the proliferation and osteogenic differentiation of AD-MSCs but inhibited cell viability and chondrogenic differentiation.	[184]
		2020	Li et al.	Biochem Biophys Res Commun.	TGF-*β*3 (100 ng/mL) promoted the chondrogenic differentiation of AD-MSCs.	[198]
		2020	Archacka et al.	Cells	IL-4 (10 ng/mL) enhanced the migration ability of AD-MSCs.	[199]
		2020	Zimowska et al.	Int J Mol Sci	IL-4 (10 ng/mL) promoted AD-MSC proliferation and migration.	[200]
	NP-MSCs	2015	Tao et al.	Growth Factors	TGF-*β*3 (10 ng/mL) combined with IGF-1 (10 ng/mL) promoted NP-MSC viability and differentiation towards NP-like phenotype.	[195]
		2019	Cheng et al.	J Cell Biochem	High concentrations of TNF-*α* (50-200 ng/mL) induced apoptosis of NP-MSCs, whereas a relatively low concentrations of TNF-*α* (0.1-10 ng/mL) promoted NP-MSC proliferation and migration but inhibited their differentiation into NP-like cells.	[186]
	UC-MSCs	2018	Yang et al.	Mol Cell Biochem	IL-1*β* (20 ng/mL) and TNF-*α* (20 ng/mL) inhibited the proliferation of UC-MSCs but promoted their chondrogenic differentiation, and IL-6 (20 ng/mL) inhibited the chondrogenic differentiation of UC-MSCs.	[187]
	PMSCs	2007	Li et al.	Cells Tissues Organs	Proinflammatory cytokines (IL-1, IL-6, and IL-8) promoted the proliferation of PMSCs in a dose-dependent manner, while anti-inflammatory cytokine (IL-4) inhibited the proliferation of PMSCs in a dose-dependent manner.	[201]
		2018	Yi et al.	Cellular Immunology	IFN-*γ* (20 ng/mL) inhibited the proliferation and migration of PMSCs.	[203]
		2020	Zhang et al.	Cell Immunol	IL-1*β* (20 ng/mL or 30 ng/mL) promoted the migration of PMSCs but inhibited cell proliferation.	[202]
	AF-MSCs	2011	Park et al.	Biomaterials	TGF-*β*3 (100 ng/mL) promoted the proliferation and chondrogenic differentiation of AF-MSCs.	[205]
	AMSCs	2019	Borem et al.	J Orthop Res	Compared with AD-MSCS, the same inflammation conditions promoted chondrogenic differentiation of AMSC.	[207]
	PB-MSCs	2018	Calle et al.	Stem Cell Res Ther	IL-1*β* promoted the migration of AD-MSCs and PB-MSCs.	[206]

MMP	BMSCs	2006	Neth et al.	Stem Cells	MT1-MMP promoted BMSC proliferation and migration.	[210]
		2007	Ries et al.	Blood	MMP-2, MT1-MMP, and TIMP-2 promoted BMSC migration, while TIMP-1 inhibited their migration.	[211]
		2010	Lu et al.	Blood	MT1-MMP dominated BMSC migration and differentiation into NP-like cells.	[212]
		2013	Sun et al.	Cell Signal	MT1-MMP promoted BMSC proliferation.	[213]
		2016	Gao et al.	Mol Reprod Dev	Silencing MMP-2 reduced BMSC proliferation and migration.	[214]
	AD-MSCs	2019	He et al.	Am J Physiol Heart Circ Physiol	MMP-9 promoted AD-MSC proliferation and migration.	[215]
		2020	Rong et al.	Dermatol Ther	Overexpression of MMP-3 reduced the expression level of collagen-I in AD-MSCs.	[216]
	NP-MSCs	2019	Zhang et al.	Cell Signal	Downregulation of MMP-3 promoted the chondrogenic differentiation of NP-MSCs.	[217]
	UC-MSCs	2014	Marquez-Curtis et al.	Stem Cells Int	Increased expression of MMP-2 promoted UC-MSC migration.	[218]

AD-MSCs: adipose-derived mesenchymal stem cells; AF-MSCs: amniotic fluid-derived mesenchymal stem cells; AMSCs: amniotic membrane-derived mesenchymal stem cells; BMSCs: bone marrow-derived mesenchymal stem cells; GDF: growth and differentiation factor; IL: interleukin; IVD: intervertebral disc; MMP: matrix metalloproteinases; MT1-MMP: membrane-type matrix metalloproteinases; NP-MSCs: nucleus pulposus-derived mesenchymal stem cells; PB-MSCs: peripheral blood-derived mesenchymal stem cells; PMSCs: placenta-derived mesenchymal stem cells; TGF: transforming growth factor; TIMPs: tissue inhibitors of metalloproteinases; TNF-*α*: tumor necrosis factor-*α*; UC-MSCs: umbilical cord-derived mesenchymal stem cells.

## Data Availability

Data sharing is not applicable to this article as no new data was created or analyzed in this study.

## References

[1] Knezevic N. N., Candido K. D., Vlaeyen J. W. S., Van Zundert J., Cohen S. P. (2021). Low back pain. *The Lancet*.

[2] Fatoye F., Gebrye T., Odeyemi I. (2019). Real-world incidence and prevalence of low back pain using routinely collected data. *Rheumatology International*.

[3] Hurwitz E. L., Randhawa K., Yu H., Côté P., Haldeman S. (2018). The global spine care initiative: a summary of the global burden of low back and neck pain studies. *European Spine Journal*.

[4] Battié M. C., Joshi A. B., Gibbons L. E. (2019). Degenerative disc disease: what is in a name?. *Spine*.

[5] Fujii K., Yamazaki M., Kang J. D. (2019). Discogenic back pain: literature review of definition, diagnosis, and treatment. *JBMR Plus*.

[6] Tan J. H., Li Z. P., Liu L. L., Liu H., Xue J. B. (2022). IL-17 in intervertebral disc degeneration: mechanistic insights and therapeutic implications. *Cell Biology International*.

[7] Yang S., Boudier-Revéret M., Chang M. C. (2021). Use of pulsed radiofrequency for the treatment of discogenic back pain: a narrative review. *Pain Practice*.

[8] Michalik A. J., Patel R. K. (2021). Evaluation of transforaminal epidural steroid injections for discogenic axial lumbosacral back pain utilizing promis as an outcome measure. *The Spine Journal*.

[9] Pan M., Li Q., Li S. (2020). Percutaneous endoscopic lumbar discectomy: indications and complications. *Pain Physician*.

[10] Kim H. S., Raorane H. D., Wu P. H., Heo D. H., Sharma S. B., Jang I. T. (2020). Incidental durotomy during endoscopic stenosis lumbar decompression: incidence, classification, and proposed management strategies. *World Neurosurgery*.

[11] Liu X. Y., Yang L. P., Zhao L. (2020). Stem cell therapy for Alzheimer's disease. *World Journal of Stem Cells*.

[12] Protze S. I., Lee J. H., Keller G. M. (2019). Human pluripotent stem cell-derived cardiovascular cells: from developmental biology to therapeutic applications. *Cell Stem Cell*.

[13] Chen S., Du K., Zou C. (2020). Current progress in stem cell therapy for type 1 diabetes mellitus. *Stem Cell Research & Therapy*.

[14] Oehme D., Goldschlager T., Ghosh P., Rosenfeld J. V., Jenkin G. (2015). Cell-based therapies used to treat lumbar degenerative disc disease: a systematic review of animal studies and human clinical trials. *Stem cells International*.

[15] Xiao L., Xu S. J., Liu C., Wang J., Hu B., Xu H. G. (2021). Sod2 and catalase improve pathological conditions of intervertebral disc degeneration by modifying human adipose-derived mesenchymal stem cells. *Life Sciences*.

[16] Wang Z., Perez-Terzic C. M., Smith J. (2015). Efficacy of intervertebral disc regeneration with stem cells - a systematic review and meta-analysis of animal controlled trials. *Gene*.

[17] Zhang W., Sun T., Li Y. (2022). Application of stem cells in the repair of intervertebral disc degeneration. *Stem Cell Research & Therapy*.

[18] Kumar H., Ha D. H., Lee E. J. (2017). Safety and tolerability of intradiscal implantation of combined autologous adipose-derived mesenchymal stem cells and hyaluronic acid in patients with chronic discogenic low back pain: 1-year follow-up of a phase i study. *Stem Cell Research & Therapy*.

[19] Mochida J., Sakai D., Nakamura Y., Watanabe T., Yamamoto Y., Kato S. (2015). Intervertebral disc repair with activated nucleus pulposus cell transplantation: a three-year, prospective clinical study of its safety. *European Cells and Materials*.

[20] Vadalà G., Ambrosio L., Russo F., Papalia R., Denaro V. (2019). Interaction between mesenchymal stem cells and intervertebral disc microenvironment: from cell therapy to tissue engineering. *Stem Cells International*.

[21] Huang Y. C., Leung V. Y., Lu W. W., Luk K. D. (2013). The effects of microenvironment in mesenchymal stem cell-based regeneration of intervertebral disc. *The Spine Journal*.

[22] Matta A., Karim M. Z., Gerami H., Benigno B., Erwin W. M. (2021). A comparative study of mesenchymal stem cell transplantation and NTG-101 molecular therapy to treat degenerative disc disease. *Scientific Reports*.

[23] Ma K., Chen S., Li Z. (2019). Mechanisms of endogenous repair failure during intervertebral disc degeneration. *Osteoarthritis and Cartilage*.

[24] Krut Z., Pelled G., Gazit D., Gazit Z. (2021). Stem cells and exosomes: new therapies for intervertebral disc degeneration. *Cells*.

[25] Yang S., Zhang F., Ma J., Ding W. (2020). Intervertebral disc ageing and degeneration: the antiapoptotic effect of oestrogen. *Ageing Research Reviews*.

[26] Wei Q., Zhang X., Zhou C., Ren Q., Zhang Y. (2019). Roles of large aggregating proteoglycans in human intervertebral disc degeneration. *Connective Tissue Research*.

[27] Ehlicke F., Freimark D., Heil B., Dorresteijn A., Czermak P. (2010). Intervertebral disc regeneration: influence of growth factors on differentiation of human mesenchymal stem cells (hmsc). *The International Journal of Artificial Organs*.

[28] Le Maitre C. L., Baird P., Freemont A. J., Hoyland J. A. (2009). An in vitro study investigating the survival and phenotype of mesenchymal stem cells following injection into nucleus pulposus tissue. *Arthritis Research & Therapy*.

[29] Bianco P. (2014). “Mesenchymal” stem cells. *Annual Review of Cell and Developmental Biology*.

[30] Caplan A. I. (2007). Adult mesenchymal stem cells for tissue engineering versus regenerative medicine. *Journal of Cellular Physiology*.

[31] Bertolo A., Thiede T., Aebli N., Baur M., Ferguson S. J., Stoyanov J. V. (2011). Human mesenchymal stem cell co-culture modulates the immunological properties of human intervertebral disc tissue fragments in vitro. *European Spine Journal*.

[32] Shim E. K., Lee J. S., Kim D. E. (2016). Autogenous mesenchymal stem cells from the vertebral body enhance intervertebral disc regeneration via paracrine interaction: an in vitro pilot study. *Cell Transplantation*.

[33] Croft A. S., Illien-Jünger S., Grad S., Guerrero J., Wangler S., Gantenbein B. (2021). The application of mesenchymal stromal cells and their homing capabilities to regenerate the intervertebral disc. *International Journal of Molecular Sciences*.

[34] Fong E. L., Chan C. K., Goodman S. B. (2011). Stem cell homing in musculoskeletal injury. *Biomaterials*.

[35] Henriksson H., Thornemo M., Karlsson C. (2009). Identification of cell proliferation zones, progenitor cells and a potential stem cell niche in the intervertebral disc region: a study in four species. *Spine*.

[36] Wang H., Zhou Y., Chu T. W. (2016). Distinguishing characteristics of stem cells derived from different anatomical regions of human degenerated intervertebral discs. *European Spine Journal*.

[37] Liu S., Liang H., Lee S. M., Li Z., Zhang J., Fei Q. (2017). Isolation and identification of stem cells from degenerated human intervertebral discs and their migration characteristics. *Acta Biochimica et Biophysica Sinica*.

[38] Blanco J. F., Graciani I. F., Sanchez-Guijo F. M. (2010). Isolation and characterization of mesenchymal stromal cells from human degenerated nucleus pulposus: comparison with bone marrow mesenchymal stromal cells from the same subjects. *Spine*.

[39] Liu L. T., Huang B., Li C. Q., Zhuang Y., Wang J., Zhou Y. (2011). Characteristics of stem cells derived from the degenerated human intervertebral disc cartilage endplate. *PLoS One*.

[40] Liu J., Tao H., Wang H. (2017). Biological behavior of human nucleus pulposus mesenchymal stem cells in response to changes in the acidic environment during intervertebral disc degeneration. *Stem Cells and Development*.

[41] Huang B., Liu L. T., Li C. Q. (2012). Study to determine the presence of progenitor cells in the degenerated human cartilage endplates. *European Spine Journal*.

[42] Sakai D., Nakamura Y., Nakai T. (2012). Exhaustion of nucleus pulposus progenitor cells with ageing and degeneration of the intervertebral disc. *Nature Communications*.

[43] Liang L., Li X., Li D. (2017). The characteristics of stem cells in human degenerative intervertebral disc. *Medicine*.

[44] Li H., Tao Y., Liang C. (2013). Influence of hypoxia in the intervertebral disc on the biological behaviors of rat adipose- and nucleus pulposus-derived mesenchymal stem cells. *Cells Tissues Organs*.

[45] Li X., Wang M., Jing X. (2018). Bone marrow- and adipose tissue-derived mesenchymal stem cells: characterization, differentiation, and applications in cartilage tissue engineering. *Critical Reviews in Eukaryotic Gene Expression*.

[46] Li X. C., Tang Y., Wu J. H., Yang P. S., Wang D. L., Ruan D. K. (2017). Characteristics and potentials of stem cells derived from human degenerated nucleus pulposus: potential for regeneration of the intervertebral disc. *BMC Musculoskeletal Disorders*.

[47] Teunissen M., Verseijden F., Riemers F. M., van Osch G., Tryfonidou M. A. (2021). The lower in vitro chondrogenic potential of canine adipose tissue-derived mesenchymal stromal cells (MSC) compared to bone marrow-derived MSC is not improved by BMP-2 or BMP-6. *The Veterinary Journal*.

[48] Wankhade U. D., Shen M., Kolhe R., Fulzele S. (2016). Advances in adipose-derived stem cells isolation, characterization, and application in regenerative tissue engineering. *Stem Cells International*.

[49] Zeckser J., Wolff M., Tucker J., Goodwin J. (2016). Multipotent mesenchymal stem cell treatment for discogenic low back pain and disc degeneration. *Stem Cells International*.

[50] Ekram S., Khalid S., Bashir I., Salim A., Khan I. (2021). Human umbilical cord-derived mesenchymal stem cells and their chondroprogenitor derivatives reduced pain and inflammation signaling and promote regeneration in a rat intervertebral disc degeneration model. *Molecular and Cellular Biochemistry*.

[51] Zhao Y. T., Qin Y., Yang J. S. (2020). Wharton's jelly-derived mesenchymal stem cells suppress apoptosis of nucleus pulposus cells in intervertebral disc degeneration via wnt pathway. *European Review for Medical and Pharmacological Sciences*.

[52] Sun Z., Tang X., Li Q., Wang H., Sun H., Tian J. (2022). Mesenchymal stem cell extracellular vesicles-derived microRNA-194-5p delays the development of intervertebral disc degeneration by targeting TRAF6. *Regenerative Therapy*.

[53] Doyle L. M., Wang M. Z. (2019). Overview of extracellular vesicles, their origin, composition, purpose, and methods for exosome isolation and analysis. *Cells*.

[54] Malekpour K., Hazrati A., Zahar M. (2022). The potential use of mesenchymal stem cells and their derived exosomes for orthopedic diseases treatment. *Stem Cell Reviews and Reports*.

[55] Valadi H., Ekström K., Bossios A., Sjöstrand M., Lee J. J., Lötvall J. O. (2007). Exosome-mediated transfer of mrnas and micrornas is a novel mechanism of genetic exchange between cells. *Nature Cell Biology*.

[56] Patel G. K., Khan M. A., Zubair H. (2019). Comparative analysis of exosome isolation methods using culture supernatant for optimum yield, purity and downstream applications. *Scientific Reports*.

[57] Yeo R. W., Lai R. C., Zhang B. (2013). Mesenchymal stem cell: an efficient mass producer of exosomes for drug delivery. *Advanced Drug Delivery Reviews*.

[58] Bhujel B., Shin H. E., Choi D. J., Han I. (2022). Mesenchymal stem cell-derived exosomes and intervertebral disc regeneration: review. *International Journal of Molecular Sciences*.

[59] Zhang J., Zhang J., Zhang Y. (2020). Mesenchymal stem cells-derived exosomes ameliorate intervertebral disc degeneration through inhibiting pyroptosis. *Journal of Cellular and Molecular Medicine*.

[60] Xia C., Zeng Z., Fang B. (2019). Mesenchymal stem cell-derived exosomes ameliorate intervertebral disc degeneration via anti-oxidant and anti-inflammatory effects. *Free Radical Biology & Medicine*.

[61] Hingert D., Ekström K., Aldridge J., Crescitelli R., Brisby H. (2020). Correction to: Extracellular vesicles from human mesenchymal stem cells expedite chondrogenesis in 3d human degenerative disc cell cultures. *Stem Cell Research & Therapy*.

[62] Marbán E. (2018). The secret life of exosomes: what bees can teach us about next-generation therapeutics. *Journal of the American College of Cardiology*.

[63] Shahabipour F., Banach M., Sahebkar A. (2016). Exosomes as nanocarriers for sirna delivery: paradigms and challenges. *Archives of Medical Science*.

[64] Zhang Y., Liu Y., Liu H., Tang W. H. (2019). Exosomes: biogenesis, biologic function and clinical potential. *Cell & Bioscience*.

[65] Li X., Corbett A. L., Taatizadeh E. (2019). Challenges and opportunities in exosome research-perspectives from biology, engineering, and cancer therapy. *APL Bioengineering*.

[66] Wei W., Ao Q., Wang X. (2020). Mesenchymal stem cell-derived exosomes: a promising biological tool in nanomedicine. *Frontiers in Pharmacology*.

[67] Ayers L., Pink R., Carter D. R. F., Nieuwland R. (2019). Clinical requirements for extracellular vesicle assays. *Journal of Extracellular Vesicles*.

[68] Forsberg M. H., Kink J. A., Hematti P., Capitini C. M. (2020). Mesenchymal stromal cells and exosomes: progress and challenges. *Frontiers in Cell and Developmental Biology*.

[69] Guerrero J., Häckel S., Croft A. S., Hoppe S., Albers C. E., Gantenbein B. (2021). The nucleus pulposus microenvironment in the intervertebral disc: the fountain of youth?. *European Cells and Materials*.

[70] Lyu F. J., Cheung K. M., Zheng Z., Wang H., Sakai D., Leung V. Y. (2019). Ivd progenitor cells: a new horizon for understanding disc homeostasis and repair. *Nature Reviews Rheumatology*.

[71] Yin X., Motorwala A., Vesvoranan O., Levene H. B., Gu W., Huang C. Y. (2020). Effects of glucose deprivation on atp and proteoglycan production of intervertebral disc cells under hypoxia. *Scientific Reports*.

[72] Grayson W. L., Zhao F., Izadpanah R., Bunnell B., Ma T. (2006). Effects of hypoxia on human mesenchymal stem cell expansion and plasticity in 3d constructs. *Journal of Cellular Physiology*.

[73] Grayson W. L., Zhao F., Bunnell B., Ma T. (2007). Hypoxia enhances proliferation and tissue formation of human mesenchymal stem cells. *Biochemical and Biophysical Research Communications*.

[74] Tsai C. C., Chen Y. J., Yew T. L. (2011). Hypoxia inhibits senescence and maintains mesenchymal stem cell properties through down-regulation of E2A-p21 by HIF-TWIST. *Blood*.

[75] Wang W., Wang Y., Deng G. (2018). Transplantation of hypoxic-preconditioned bone mesenchymal stem cells retards intervertebral disc degeneration via enhancing implanted cell survival and migration in rats. *Stem Cells International*.

[76] Raheja L. F., Genetos D. C., Wong A., Yellowley C. E. (2011). Hypoxic regulation of mesenchymal stem cell migration: the role of RhoA and HIF-1*α*. *Cell Biology International*.

[77] Felka T., Schäfer R., Schewe B., Benz K., Aicher W. K. (2009). Hypoxia reduces the inhibitory effect of IL-1beta on chondrogenic differentiation of FCS-free expanded MSC. *Osteoarthritis and Cartilage*.

[78] Risbud M. V., Albert T. J., Guttapalli A. (2004). Differentiation of mesenchymal stem cells towards a nucleus pulposus-like phenotype in vitro: implications for cell-based transplantation therapy. *Spine*.

[79] Müller J., Benz K., Ahlers M., Gaissmaier C., Mollenhauer J. (2011). Hypoxic conditions during expansion culture prime human mesenchymal stromal precursor cells for chondrogenic differentiation in three-dimensional cultures. *Cell Transplantation*.

[80] Stoyanov J. V., Gantenbein-Ritter B., Bertolo A. (2011). Role of hypoxia and growth and differentiation factor-5 on differentiation of human mesenchymal stem cells towards intervertebral nucleus pulposus-like cells. *European Cells and Materials*.

[81] Kanichai M., Ferguson D., Prendergast P. J., Campbell V. A. (2008). Hypoxia promotes chondrogenesis in rat mesenchymal stem cells: a role for AKT and hypoxia-inducible factor (HIF)-1alpha. *Journal of Cellular Physiology*.

[82] Peck S. H., Bendigo J. R., Tobias J. W. (2021). Hypoxic preconditioning enhances bone marrow-derived mesenchymal stem cell survival in a low oxygen and nutrient-limited 3d microenvironment. *Cartilage*.

[83] Hwang O. K., Noh Y. W., Hong J. T., Lee J. W. (2020). Hypoxia pretreatment promotes chondrocyte differentiation of human adipose-derived stem cells via vascular endothelial growth factor. *Tissue Engineering and Regenerative Medicine*.

[84] Portron S., Merceron C., Gauthier O. (2013). Effects of in vitro low oxygen tension preconditioning of adipose stromal cells on their in vivo chondrogenic potential: application in cartilage tissue repair. *PLoS One*.

[85] Govoni M., Muscari C., Bonafè F. (2021). A brief very-low oxygen tension regimen is sufficient for the early chondrogenic commitment of human adipose-derived mesenchymal stem cells. *Advances in Medical Sciences*.

[86] Choi J. R., Pingguan-Murphy B., Abas W. A. B. W. (2014). Hypoxia promotes growth and viability of human adipose-derived stem cells with increased growth factors secretion. *Journal of Asian Scientific Research*.

[87] Fotia C., Massa A., Boriani F., Baldini N., Granchi D. (2015). Hypoxia enhances proliferation and stemness of human adipose-derived mesenchymal stem cells. *Cytotechnology*.

[88] Wan Safwani W. K. Z., Choi J. R., Yong K. W., Ting I., Mat Adenan N. A., Pingguan-Murphy B. (2017). Hypoxia enhances the viability, growth and chondrogenic potential of cryopreserved human adipose-derived stem cells. *Cryobiology*.

[89] Takahashi A., Nakajima H., Uchida K. (2018). Comparison of mesenchymal stromal cells isolated from murine adipose tissue and bone marrow in the treatment of spinal cord injury. *Cell Transplantation*.

[90] Deng Y., Huang G., Chen F. (2019). Hypoxia enhances buffalo adipose-derived mesenchymal stem cells proliferation, stemness, and reprogramming into induced pluripotent stem cells. *Journal of Cellular Physiology*.

[91] Chung D. J., Hayashi K., Toupadakis C. A., Wong A., Yellowley C. E. (2012). Osteogenic proliferation and differentiation of canine bone marrow and adipose tissue derived mesenchymal stromal cells and the influence of hypoxia. *Research in Veterinary Science*.

[92] He R., Wang Z., Cui M. (2021). HIF1A alleviates compression-induced apoptosis of nucleus pulposus derived stem cells via upregulating autophagy. *Autophagy*.

[93] Lee H. J., Ryu J. M., Jung Y. H., Oh S. Y., Lee S. J., Han H. J. (2015). Novel pathway for hypoxia-induced proliferation and migration in human mesenchymal stem cells: involvement of HIF-1*α*, FASN, and mTORC1. *Stem Cells*.

[94] Yang H., Ni L., Liu X. (2012). Effects of hypoxia on differentiation from human placenta-derived mesenchymal stem cells to nucleus pulposus-like cells. *The Spine Journal*.

[95] Ni L., Liu X., Sochacki K. R. (2014). Effects of hypoxia on differentiation from human placenta-derived mesenchymal stem cells to nucleus pulposus-like cells. *The Spine Journal*.

[96] Choi J. H., Lim S. M., Yoo Y. I., Jung J., Park J. W., Kim G. J. (2016). Microenvironmental interaction between hypoxia and endothelial cells controls the migration ability of placenta-derived mesenchymal stem cells via *α*4 integrin and rho signaling. *Journal of Cellular Biochemistry*.

[97] Li L., Jaiswal P. K., Makhoul G. (2017). Hypoxia modulates cell migration and proliferation in placenta-derived mesenchymal stem cells. *The Journal of Thoracic and Cardiovascular Surgery*.

[98] Kwon S. Y., Chun S. Y., Ha Y. S. (2017). Hypoxia enhances cell properties of human mesenchymal stem cells. *Tissue Engineering and Regenerative Medicine*.

[99] Casciaro F., Borghesan M., Beretti F. (2020). Prolonged hypoxia delays aging and preserves functionality of human amniotic fluid stem cells. *Mechanisms of Ageing and Development*.

[100] Wang P., Zhu P., Yu C., Wu J. (2022). The proliferation and stemness of peripheral blood-derived mesenchymal stromal cells were enhanced by hypoxia. *Frontiers in endocrinology*.

[101] Silva J. C., Han X., Silva T. P. (2020). Glycosaminoglycan remodeling during chondrogenic differentiation of human bone marrow-/synovial-derived mesenchymal stem/stromal cells under normoxia and hypoxia. *Glycoconjugate Journal*.

[102] Shirazi-Adl A., Taheri M., Urban J. P. (2010). Analysis of cell viability in intervertebral disc: effect of endplate permeability on cell population. *Journal of Biomechanics*.

[103] Guehring T., Wilde G., Sumner M. (2009). Notochordal intervertebral disc cells: sensitivity to nutrient deprivation. *Arthritis and Rheumatism*.

[104] Wuertz K., Godburn K., Neidlinger-Wilke C., Urban J., Iatridis J. C. (2008). Behavior of mesenchymal stem cells in the chemical microenvironment of the intervertebral disc. *Spine*.

[105] Liang C., Li H., Tao Y. (2012). Responses of human adipose-derived mesenchymal stem cells to chemical microenvironment of the intervertebral disc. *Journal of Translational Medicine*.

[106] Jünger S., Gantenbein-Ritter B., Lezuo P., Alini M., Ferguson S. J., Ito K. (2009). Effect of limited nutrition on in situ intervertebral disc cells under simulated-physiological loading. *Spine*.

[107] Farrell M. J., Shin J. I., Smith L. J., Mauck R. L. (2015). Functional consequences of glucose and oxygen deprivation on engineered mesenchymal stem cell-based cartilage constructs. *Osteoarthritis and Cartilage*.

[108] Naqvi S. M., Buckley C. T. (2015). Extracellular matrix production by nucleus pulposus and bone marrow stem cells in response to altered oxygen and glucose microenvironments. *Journal of Anatomy*.

[109] Yang Z., Qi Z., Yang X., Gao Q., Hu Y., Yuan X. (2022). Inhibition of RIP3 increased ADSC viability under OGD and modified the competency of adipogenesis, angiogenesis, and inflammation regulation. *Bioscience Reports*.

[110] Parker A., Shang H., Khurgel M., Katz A. (2007). Low serum and serum-free culture of multipotential human adipose stem cells. *Cytotherapy*.

[111] Wan Safwani W. K., Wong C. W., Yong K. W. (2016). The effects of hypoxia and serum-free conditions on the stemness properties of human adipose-derived stem cells. *Cytotechnology*.

[112] Potier E., Ferreira E., Meunier A., Sedel L., Logeart-Avramoglou D., Petite H. (2007). Prolonged hypoxia concomitant with serum deprivation induces massive human mesenchymal stem cell death. *Tissue Engineering*.

[113] Pang H., Zhou Y., Wang J. (2021). Berberine influences the survival of fat grafting by inhibiting autophagy and apoptosis of human adipose derived mesenchymal stem cells. *Drug Design Development and Therapy*.

[114] Ghorbani A., Baradaran Rahimi V., Sadeghnia H. R., Hosseini A. (2018). Effect of berberine on the viability of adipose tissue-derived mesenchymal stem cells in nutrients deficient condition. *Natural Product Research*.

[115] Abdolmaleki A., Ghayour M. B., Behnam-Rassouli M. (2020). Protective effects of acetyl-l-carnitine against serum and glucose deprivation-induced apoptosis in rat adipose-derived mesenchymal stem cells. *Cell and Tissue Banking*.

[116] Pan T., Qian Y., Li T. (2022). Acetyl l-carnitine protects adipose-derived stem cells against serum-starvation: regulation on the network composed of reactive oxygen species, autophagy, apoptosis and senescence. *Cytotechnology*.

[117] Huang Y. C., Yang Z. M., Chen X. H. (2009). Isolation of mesenchymal stem cells from human placental decidua basalis and resistance to hypoxia and serum deprivation. *Stem Cell Reviews and Reports*.

[118] Huang Y. C., Yang Z. M., Jiang N. G. (2010). Characterization of MSCs from human placental decidua basalis in hypoxia and serum deprivation. *Cell Biology International*.

[119] He J., Wang C., Sun Y. (2016). Exendin-4 protects bone marrow-derived mesenchymal stem cells against oxygen/glucose and serum deprivation-induced apoptosis through the activation of the cAMP/PKA signaling pathway and the attenuation of ER stress. *International Journal of Molecular Medicine*.

[120] Liu D., Tang W., Zhang H. (2020). Icariin protects rabbit BMSCs against OGD-induced apoptosis by inhibiting ERs-mediated autophagy via MAPK signaling pathway. *Life Sciences*.

[121] Wu F., Ye H., Lin J. (2018). TGF-*β*3 reduces apoptosis in ischemia-induced adipose-derived stem cells by enhancing DNA repair. *Experimental and Therapeutic Medicine*.

[122] Li C., Chen K., Jia M. (2018). Ampk promotes survival and adipogenesis of ischemia-challenged adscs in an autophagy-dependent manner. *Biochimica et Biophysica Acta (BBA)-Molecular and Cell Biology of Lipids*.

[123] Tian D., Liu J., Chen L., Zhu B., Jing J. (2020). The protective effects of PI3K/Akt pathway on human nucleus pulposus mesenchymal stem cells against hypoxia and nutrition deficiency. *Journal of Orthopaedic Surgery and Research*.

[124] Wuertz K., Godburn K., Iatridis J. C. (2009). MSC response to pH levels found in degenerating intervertebral discs. *Biochemical and Biophysical Research Communications*.

[125] Li H., Liang C., Tao Y. (2012). Acidic pH conditions mimicking degenerative intervertebral discs impair the survival and biological behavior of human adipose-derived mesenchymal stem cells. *Experimental Biology and Medicine*.

[126] Han B., Wang H. C., Li H. (2014). Nucleus pulposus mesenchymal stem cells in acidic conditions mimicking degenerative intervertebral discs give better performance than adipose tissue-derived mesenchymal stem cells. *Cells Tissues Organs*.

[127] Naqvi S. M., Buckley C. T. (2016). Bone marrow stem cells in response to intervertebral disc-like matrix acidity and oxygen concentration: implications for cell-based regenerative therapy. *Spine*.

[128] Li X., Wu F. R., Xu R. S. (2014). Acid-sensing ion channel 1a-mediated calcium influx regulates apoptosis of endplate chondrocytes in intervertebral discs. *Expert Opinion on Therapeutic Targets*.

[129] Cuesta A., Del Valle M. E., García-Suárez O. (2014). Acid-sensing ion channels in healthy and degenerated human intervertebral disc. *Connective Tissue Research*.

[130] Yermolaieva O., Leonard A. S., Schnizler M. K., Abboud F. M., Welsh M. J. (2004). Extracellular acidosis increases neuronal cell calcium by activating acid-sensing ion channel 1a. *Proceedings of the National Academy of Sciences of the United States of America*.

[131] Swain S. M., Parameswaran S., Sahu G., Verma R. S., Bera A. K. (2012). Proton-gated ion channels in mouse bone marrow stromal cells. *Stem Cell Research*.

[132] Cai F., Hong X., Tang X. (2019). ASIC1a activation induces calcium-dependent apoptosis of BMSCs under conditions that mimic the acidic microenvironment of the degenerated intervertebral disc. *Bioscience Reports*.

[133] Gansau J., Buckley C. T. (2021). Priming as a strategy to overcome detrimental pH effects on cells for intervertebral disc regeneration. *European Cells and Materials*.

[134] Ding J., Zhang R., Li H. (2021). ASIC1 and ASIC3 mediate cellular senescence of human nucleus pulposus mesenchymal stem cells during intervertebral disc degeneration. *Aging*.

[135] Wang Z., Han L., Chen H. (2022). Sa12b improves biological activity of human degenerative nucleus pulposus mesenchymal stem cells in a severe acid environment by inhibiting acid-sensitive ion channels. *Frontiers in Bioengineering and Biotechnology*.

[136] van Dijk B., Potier E., Ito K. (2011). Culturing bovine nucleus pulposus explants by balancing medium osmolarity. *Tissue Engineering Part C, Methods*.

[137] Johnson Z. I., Shapiro I. M., Risbud M. V. (2014). Extracellular osmolarity regulates matrix homeostasis in the intervertebral disc and articular cartilage: evolving role of tonebp. *Matrix Biology*.

[138] Sadowska A., Kameda T., Krupkova O., Wuertz-Kozak K. (2018). Osmosensing, osmosignalling and inflammation: how intervertebral disc cells respond to altered osmolarity. *European Cells & Materials*.

[139] Tao Y. Q., Liang C. Z., Li H. (2013). Potential of co-culture of nucleus pulposus mesenchymal stem cells and nucleus pulposus cells in hyperosmotic microenvironment for intervertebral disc regeneration. *Cell Biology International*.

[140] Potočar U., Hudoklin S., Kreft M. E., Završnik J., Božikov K., Fröhlich M. (2016). Adipose-derived stem cells respond to increased osmolarities. *PLoS One*.

[141] Caron M. M., van der Windt A. E., Emans P. J., van Rhijn L. W., Jahr H., Welting T. J. (2013). Osmolarity determines the *in vitro* chondrogenic differentiation capacity of progenitor cells *via* nuclear factor of activated T-cells 5. *Bone*.

[142] Ahmadyan S., Kabiri M., Hanaee-Ahvaz H., Farazmand A. (2018). Osmolyte type and the osmolarity level affect chondrogenesis of mesenchymal stem cells. *Applied Biochemistry and Biotechnology*.

[143] Ahmadyan S., Kabiri M., Tasharofi N. (2018). The osmolyte type affects cartilage associated pathologic marker expression during in vitro mesenchymal stem cell chondrogenesis under hypertonic conditions. *Cellular and Molecular Biology*.

[144] Zhang Y., Wang Y., Zhou X. (2020). Osmolarity controls the differentiation of adipose-derived stem cells into nucleus pulposus cells via histone demethylase KDM4B. *Molecular and Cellular Biochemistry*.

[145] Alinezhad-Bermi S., Kabiri M., Rad I., Irani S., Hanaee-Ahvaz H. (2021). Hyperosmolarity benefits cartilage regeneration by enhancing expression of chondrogenic markers and reducing inflammatory markers. *In Vitro Cellular & Developmental Biology-Animal*.

[146] Li H., Wang J., Li F., Chen G., Chen Q. (2018). The influence of hyperosmolarity in the intervertebral disc on the proliferation and chondrogenic differentiation of nucleus pulposus-derived mesenchymal stem cells. *Cells Tissues Organs*.

[147] Gao X., Zhu Q., Gu W. (2016). Prediction of glycosaminoglycan synthesis in intervertebral disc under mechanical loading. *Journal of Biomechanics*.

[148] Gullbrand S. E., Peterson J., Mastropolo R. (2015). Low rate loading-induced convection enhances net transport into the intervertebral disc in vivo. *The Spine Journal*.

[149] Fearing B. V., Hernandez P. A., Setton L. A., Chahine N. O. (2018). Mechanotransduction and cell biomechanics of the intervertebral disc. *JOR Spine*.

[150] Angele P., Schumann D., Angele M. (2004). Cyclic, mechanical compression enhances chondrogenesis of mesenchymal progenitor cells in tissue engineering scaffolds. *Biorheology*.

[151] Huang C. Y., Reuben P. M., Cheung H. S. (2005). Temporal expression patterns and corresponding protein inductions of early responsive genes in rabbit bone marrow-derived mesenchymal stem cells under cyclic compressive loading. *Stem Cells*.

[152] Huang C. Y., Hagar K. L., Frost L. E., Sun Y., Cheung H. S. (2004). Effects of cyclic compressive loading on chondrogenesis of rabbit bone-marrow derived mesenchymal stem cells. *Stem Cells*.

[153] Mouw J. K., Connelly J. T., Wilson C. G., Michael K. E., Levenston M. E. (2007). Dynamic compression regulates the expression and synthesis of chondrocyte-specific matrix molecules in bone marrow stromal cells. *Stem Cells*.

[154] Gan Y., Tu B., Li P. (2018). Low magnitude of compression enhances biosynthesis of mesenchymal stem cells towards nucleus pulposus cells via the TRPV4-dependent pathway. *Stem Cells International*.

[155] Zhang Y., Tang C. L., Chen W. J., Zhang Q., Wang S. L. (2015). Dynamic compression combined with exogenous sox-9 promotes chondrogenesis of adipose-derived mesenchymal stem cells in plga scaffold. *European Review for Medical and Pharmacological Sciences*.

[156] Liang H., Chen S., Huang D., Deng X., Ma K., Shao Z. (2018). Effect of compression loading on human nucleus pulposus-derived mesenchymal stem cells. *Stem Cells International*.

[157] Li Z., Yao S. J., Alini M., Stoddart M. J. (2010). Chondrogenesis of human bone marrow mesenchymal stem cells in fibrin-polyurethane composites is modulated by frequency and amplitude of dynamic compression and shear stress. *Tissue Engineering Part A*.

[158] Li Z., Kupcsik L., Yao S. J., Alini M., Stoddart M. J. (2010). Mechanical load modulates chondrogenesis of human mesenchymal stem cells through the TGF-beta pathway. *Journal of Cellular and Molecular Medicine*.

[159] Angele P., Yoo J. U., Smith C. (2003). Cyclic hydrostatic pressure enhances the chondrogenic phenotype of human mesenchymal progenitor cells differentiated in vitro. *Journal of Orthopaedic Research*.

[160] Miyanishi K., Trindade M. C., Lindsey D. P. (2006). Dose- and time-dependent effects of cyclic hydrostatic pressure on transforming growth factor-beta3-induced chondrogenesis by adult human mesenchymal stem cells in vitro. *Tissue Engineering*.

[161] Ogawa R., Mizuno S., Murphy G. F., Orgill D. P. (2009). The effect of hydrostatic pressure on three-dimensional chondroinduction of human adipose-derived stem cells. *Tissue Engineering Part A*.

[162] Dai J., Wang H., Liu G., Xu Z., Li F., Fang H. (2014). Dynamic compression and co-culture with nucleus pulposus cells promotes proliferation and differentiation of adipose-derived mesenchymal stem cells. *Journal of Biomechanics*.

[163] Cheng B., Feng F., Shi F. (2022). Distinctive roles of wnt signaling in chondrogenic differentiation of bmscs under coupling of pressure and platelet-rich fibrin. *Tissue Engineering and Regenerative Medicine*.

[164] Connelly J. T., Vanderploeg E. J., Mouw J. K., Wilson C. G., Levenston M. E. (2010). Tensile loading modulates bone marrow stromal cell differentiation and the development of engineered fibrocartilage constructs. *Tissue Engineering Part A*.

[165] Baker B. M., Shah R. P., Huang A. H., Mauck R. L. (2011). Dynamic tensile loading improves the functional properties of mesenchymal stem cell-laden nanofiber-based fibrocartilage. *Tissue Engineering Part A*.

[166] Abusharkh H. A., Mallah A. H., Amr M. M. (2021). Enhanced matrix production by cocultivated human stem cells and chondrocytes under concurrent mechanical strain. *In Vitro Cellular & Developmental Biology - Animal*.

[167] Capossela S., Schläfli P., Bertolo A. (2014). Degenerated human intervertebral discs contain autoantibodies against extracellular matrix proteins. *European Cells and Materials*.

[168] Wang H. Q., Samartzis D. (2014). Clarifying the nomenclature of intervertebral disc degeneration and displacement: from bench to bedside. *International Journal of Clinical and Experimental Pathology*.

[169] Sun Z., Liu B., Luo Z. J. (2020). The immune privilege of the intervertebral disc: implications for intervertebral disc degeneration treatment. *International Journal of Medical Sciences*.

[170] Di Martino A., Merlini L., Faldini C. (2013). Autoimmunity in intervertebral disc herniation: from bench to bedside. *Expert Opinion on Therapeutic Targets*.

[171] Risbud M. V., Shapiro I. M. (2014). Role of cytokines in intervertebral disc degeneration: pain and disc content. *Nature Reviews Rheumatology*.

[172] Shamji M. F., Setton L. A., Jarvis W. (2010). Proinflammatory cytokine expression profile in degenerated and herniated human intervertebral disc tissues. *Arthritis and Rheumatism*.

[173] Khan A. N., Jacobsen H. E., Khan J. (2017). Inflammatory biomarkers of low back pain and disc degeneration: a review. *Annals of the New York Academy of Sciences*.

[174] Lyu F. J., Cui H., Pan H. (2021). Painful intervertebral disc degeneration and inflammation: from laboratory evidence to clinical interventions. *Bone Research*.

[175] Zhang G. Z., Liu M. Q., Chen H. W. (2021). NF-*κ*B signalling pathways in nucleus pulposus cell function and intervertebral disc degeneration. *Cell Proliferation*.

[176] Wang K., Bao J. P., Yang S. (2016). A cohort study comparing the serum levels of pro- or anti-inflammatory cytokines in patients with lumbar radicular pain and healthy subjects. *European Spine Journal*.

[177] Djuric N., Lafeber G. C. M., Vleggeert-Lankamp C. L. A. (2020). The contradictory effect of macrophage-related cytokine expression in lumbar disc herniations: a systematic review. *European Spine Journal*.

[178] Yamamoto Y., Kokubo Y., Nakajima H., Honjoh K., Watanabe S., Matsumine A. (2022). Distribution and polarization of hematogenous macrophages associated with the progression of intervertebral disc degeneration. *Spine*.

[179] Carrero R., Cerrada I., Lledó E. (2012). IL1*β* induces mesenchymal stem cells migration and leucocyte chemotaxis through NF-*κ*B. *Stem Cell Reviews and Reports*.

[180] Teixeira G. Q., Pereira C. L., Ferreira J. R. (2018). Immunomodulation of human mesenchymal stem/stromal cells in intervertebral disc degeneration: insights from a proinflammatory/degenerative ex vivo model. *Spine*.

[181] Ponte A. L., Marais E., Gallay N. (2007). The in vitro migration capacity of human bone marrow mesenchymal stem cells: comparison of chemokine and growth factor chemotactic activities. *Stem Cells*.

[182] Wang Y., Xu J., Zhang X. (2017). TNF-*α*-induced LRG1 promotes angiogenesis and mesenchymal stem cell migration in the subchondral bone during osteoarthritis. *Cell Death & Disease*.

[183] Xie Z., Yu W., Zheng G. (2021). TNF-*α*-mediated m^6^A modification of elmo1 triggers directional migration of mesenchymal stem cell in ankylosing spondylitis. *Nature Communications*.

[184] Brandt L., Schubert S., Scheibe P. (2018). Tenogenic properties of mesenchymal progenitor cells are compromised in an inflammatory environment. *International Journal of Molecular Sciences*.

[185] Mohammadpour H., Pourfathollah A. A., Nikougoftar Zarif M., Hashemi S. M. (2016). Increasing proliferation of murine adipose tissue-derived mesenchymal stem cells by TNF-*α* plus IFN-*γ*. *Immunopharmacology and Immunotoxicology*.

[186] Cheng S., Li X., Jia Z. (2019). The inflammatory cytokine tnf-*α* regulates the biological behavior of rat nucleus pulposus mesenchymal stem cells through the NF-*κ*B signaling pathway in vitro. *Journal of Cellular Biochemistry*.

[187] Yang C., Chen Y., Li F. (2018). The biological changes of umbilical cord mesenchymal stem cells in inflammatory environment induced by different cytokines. *Molecular and Cellular Biochemistry*.

[188] Wei H., Shen G., Deng X. (2013). The role of IL-6 in bone marrow (BM)-derived mesenchymal stem cells (MSCs) proliferation and chondrogenesis. *Cell and Tissue Banking*.

[189] Kasprzycka P., Archacka K., Kowalski K. (2019). The factors present in regenerating muscles impact bone marrow-derived mesenchymal stromal/stem cell fusion with myoblasts. *Stem Cell Research & Therapy*.

[190] Huang H., Kim H. J., Chang E. J. (2009). IL-17 stimulates the proliferation and differentiation of human mesenchymal stem cells: implications for bone remodeling. *Cell Death & Differentiation*.

[191] Mojsilović S., Krstić A., Ilić V. (2011). IL-17 and FGF signaling involved in mouse mesenchymal stem cell proliferation. *Cell and Tissue Research*.

[192] Ma T., Wang X., Jiao Y. (2018). Interleukin 17 (IL-17)-induced mesenchymal stem cells prolong the survival of allogeneic skin grafts. *Annals of Transplantation*.

[193] Chen S., Liu S., Ma K., Zhao L., Lin H., Shao Z. (2019). TGF-*β* signaling in intervertebral disc health and disease. *Osteoarthritis and Cartilage*.

[194] Gantenbein-Ritter B., Benneker L. M., Alini M., Grad S. (2011). Differential response of human bone marrow stromal cells to either TGF-*β*_1_ or rhGDF-5. *European Spine Journal*.

[195] Tao Y., Zhou X., Liang C. (2015). TGF-*β*3 and IGF-1 synergy ameliorates nucleus pulposus mesenchymal stem cell differentiation towards the nucleus pulposus cell type through MAPK/ERK signaling. *Growth Factors*.

[196] Clarke L. E., McConnell J. C., Sherratt M. J., Derby B., Richardson S. M., Hoyland J. A. (2014). Growth differentiation factor 6 and transforming growth factor-beta differentially mediate mesenchymal stem cell differentiation, composition, and micromechanical properties of nucleus pulposus constructs. *Arthritis Research & Therapy*.

[197] Colombier P., Clouet J., Boyer C. (2016). TGF-*β*1 and GDF5 act synergistically to drive the differentiation of human adipose stromal cells toward nucleus pulposus-like cells. *Stem Cells*.

[198] Li D., Ma X., Zhao T. (2020). Mechanism of TGF-*β*_3_ promoting chondrogenesis in human fat stem cells. *Biochemical and Biophysical Research Communications*.

[199] Archacka K., Bem J., Brzoska E. (2020). Beneficial effect of IL-4 and SDF-1 on myogenic potential of mouse and human adipose tissue-derived stromal cells. *Cells*.

[200] Zimowska M., Archacka K., Brzoska E. (2020). IL-4 and SDF-1 increase adipose tissue-derived stromal cell ability to improve rat skeletal muscle regeneration. *International Journal of Molecular Sciences*.

[201] Li D., Wang G. Y., Dong B. H., Zhang Y. C., Wang Y. X., Sun B. C. (2007). Biological characteristics of human placental mesenchymal stem cells and their proliferative response to various cytokines. *Cells Tissues Organs*.

[202] Zhang A., Xiong Y., Xu F. (2020). IL-1*β* enhances human placenta-derived mesenchymal stromal cells ability to mediate Th1/Th2 and Th1/CD4^+^IL-10^+^ T cell balance and regulates its adhesion, proliferation and migration via PD-L1. *Cellular Immunology*.

[203] Yi J. Z., Chen Z. H., Xu F. H. (2018). Interferon-*γ* suppresses the proliferation and migration of human placenta-derived mesenchmal stromal cells and enhances their ability to induce the generation of CD4^+^CXCR5^+^Foxp3^+^Treg subset. *Cellular Immunology*.

[204] Calle A., Gutiérrez-Reinoso M., Re M. (2021). Bovine peripheral blood mscs chemotax towards inflammation and embryo implantation stimuli. *Journal of Cellular Physiology*.

[205] Park J. S., Shim M. S., Shim S. H. (2011). Chondrogenic potential of stem cells derived from amniotic fluid, adipose tissue, or bone marrow encapsulated in fibrin gels containing TGF-*β*3. *Biomaterials*.

[206] Calle A., Barrajón-Masa C., Gómez-Fidalgo E. (2018). Iberian pig mesenchymal stem/stromal cells from dermal skin, abdominal and subcutaneous adipose tissues, and peripheral blood: in vitro characterization and migratory properties in inflammation. *Stem Cell Research & Therapy*.

[207] Borem R., Madeline A., Bowman M., Gill S., Tokish J., Mercuri J. (2019). Differential effector response of amnion- and adipose-derived mesenchymal stem cells to inflammation; implications for intradiscal therapy. *Journal of Orthopaedic Research*.

[208] Almalki S. G., Agrawal D. K. (2016). Effects of matrix metalloproteinases on the fate of mesenchymal stem cells. *Stem Cell Research & Therapy*.

[209] Zhang C., Gullbrand S. E., Schaer T. P. (2020). Inflammatory cytokine and catabolic enzyme expression in a goat model of intervertebral disc degeneration. *Journal of Orthopaedic Research*.

[210] Neth P., Ciccarella M., Egea V., Hoelters J., Jochum M., Ries C. (2006). Wnt signaling regulates the invasion capacity of human mesenchymal stem cells. *Stem Cells*.

[211] Ries C., Egea V., Karow M., Kolb H., Jochum M., Neth P. (2007). MMP-2, MT1-MMP, and TIMP-2 are essential for the invasive capacity of human mesenchymal stem cells: differential regulation by inflammatory cytokines. *Blood*.

[212] Lu C., Li X. Y., Hu Y., Rowe R. G., Weiss S. J. (2010). MT1-MMP controls human mesenchymal stem cell trafficking and differentiation. *Blood*.

[213] Sun X., Gao X., Zhou L., Sun L., Lu C. (2013). PDGF-BB-induced MT1-mmp expression regulates proliferation and invasion of mesenchymal stem cells in 3-dimensional collagen via MEK/ERK1/2 and PI3K/AKT signaling. *Cellular Signalling*.

[214] Gao F., Sun M., Gong Y., Wang H., Wang Y., Hou H. (2016). MicroRNA-195a-3p inhibits angiogenesis by targeting Mmp2 in murine mesenchymal stem cells. *Molecular Reproduction and Development*.

[215] He Y., Guo Y., Xia Y. (2019). Resistin promotes cardiac homing of mesenchymal stem cells and functional recovery after myocardial ischemia-reperfusion via the ERK1/2-MMP-9 pathway. *American Journal of Physiology Heart and Circulatory Physiology*.

[216] Rong S., Li C., Li S., Wu S., Sun F. (2020). Genetically modified adipose-derived stem cells with matrix metalloproteinase 3 promote scarless cutaneous repair. *Dermatologic Therapy*.

[217] Zhang Q., Shen Y., Zhao S., Jiang Y., Zhou D., Zhang Y. (2021). Exosomes miR-15a promotes nucleus pulposus-mesenchymal stem cells chondrogenic differentiation by targeting MMP-3. *Cellular Signalling*.

[218] Marquez-Curtis L. A., Qiu Y., Xu A., Janowska-Wieczorek A. (2014). Migration, proliferation, and differentiation of cord blood mesenchymal stromal cells treated with histone deacetylase inhibitor valproic acid. *Stem Cells International*.

